# Harmonization of supervised machine learning practices for efficient source attribution of *Listeria monocytogenes* based on genomic data

**DOI:** 10.1186/s12864-023-09667-w

**Published:** 2023-09-22

**Authors:** Pierluigi Castelli, Andrea De Ruvo, Andrea Bucciacchio, Nicola D’Alterio, Cesare Cammà, Adriano Di Pasquale, Nicolas Radomski

**Affiliations:** Istituto Zooprofilattico Sperimentale dell’Abruzzo e del Molise “Giuseppe Caporale” (IZSAM), National Reference Centre (NRC) for Whole Genome Sequencing of microbial pathogens: data base and bioinformatics analysis (GENPAT), Via Campo Boario, Teramo, TE 64100 Italy

**Keywords:** Source attribution, *Listeria monocytogenes*, Machine learning, Genomic data

## Abstract

**Background:**

Genomic data-based machine learning tools are promising for real-time surveillance activities performing source attribution of foodborne bacteria such as *Listeria monocytogenes*. Given the heterogeneity of machine learning practices, our aim was to identify those influencing the source prediction performance of the usual holdout method combined with the repeated k-fold cross-validation method.

**Methods:**

A large collection of 1 100 *L.* *monocytogenes* genomes with known sources was built according to several genomic metrics to ensure authenticity and completeness of genomic profiles. Based on these genomic profiles (i.e. 7-locus alleles, core alleles, accessory genes, core SNPs and pan kmers), we developed a versatile workflow assessing prediction performance of different combinations of training dataset splitting (i.e. 50, 60, 70, 80 and 90%), data preprocessing (i.e. with or without near-zero variance removal), and learning models (i.e. BLR, ERT, RF, SGB, SVM and XGB). The performance metrics included accuracy, Cohen’s kappa, F1-score, area under the curves from receiver operating characteristic curve, precision recall curve or precision recall gain curve, and execution time.

**Results:**

The testing average accuracies from accessory genes and pan kmers were significantly higher than accuracies from core alleles or SNPs. While the accuracies from 70 and 80% of training dataset splitting were not significantly different, those from 80% were significantly higher than the other tested proportions. The near-zero variance removal did not allow to produce results for 7-locus alleles, did not impact significantly the accuracy for core alleles, accessory genes and pan kmers, and decreased significantly accuracy for core SNPs. The SVM and XGB models did not present significant differences in accuracy between each other and reached significantly higher accuracies than BLR, SGB, ERT and RF, in this order of magnitude. However, the SVM model required more computing power than the XGB model, especially for high amount of descriptors such like core SNPs and pan kmers.

**Conclusions:**

In addition to recommendations about machine learning practices for *L.* *monocytogenes* source attribution based on genomic data, the present study also provides a freely available workflow to solve other balanced or unbalanced multiclass phenotypes from binary and categorical genomic profiles of other microorganisms without source code modifications.

**Supplementary Information:**

The online version contains supplementary material available at 10.1186/s12864-023-09667-w.

## Introduction

The foodborne pathogen *Listeria monocytogenes*, responsible for human listeriosis, has become a model in infection biology during the last decades and its infection process is today almost completely understood (i.e. encounter of the host intestinal epithelium after ingestion of contaminated food, crossing of intestinal epithelial barrier into the lamina propria, dissemination through the lymph and blood towards the liver and spleen) [1]. Depending on ingested *L.* *monocytogenes* doses, immunocompetent individuals may develop mild to severe gastro-enteritis and people at risk (e.g. like children, elderly individuals, immunocompromised individuals and pregnant women) may suffer of bacterial sepsis, subsequent bacterial meningitis and/or infection of the fetus [2]. *L.* *monocytogenes* is a Gram-positive rod-shaped bacterium belonging to the genus *Listeria* encompassing 17 other species and harbors 4 main evolutionary lineages, 13 agglutination serotypes, 5 molecular serotypes, as well as several clonal complexes (CCs) and sequence types (STs) identified by multi-locus sequence typing (MLST) [3]. At the genomic scale, *L. monocytogenes* is a clonal species [3] with a small chromosome (~ 3 Mbp) [4] and a low GC content (i.e. 37–38%) [4–6]. *L.* *monocytogenes* possesses between 2 330 and 2 456 core genes [7], as well as potentially several hundreds of accessory genes [4] harbored by plasmids [8] and phages [5] of various sizes.

The global public health burden of listeriosis was estimated annually at thousands of deaths and tens of thousands disability-adjusted life-years [9]. This public health burden is accompanied by a considerable economic cost, which for example has reached hundreds million Canadian dollars for an outbreak related to contaminated delicatessen meat [10]. According to the European Union (EU) One Health 2021 Zoonoses Report, *L.* *monocytogenes* infections were the fifth most reported zoonoses in humans in 2021 and part the most severe zoonotic diseases, with the most hospitalisations and highest case fatality rates [11]. Even if *L.* *monocytogenes* may be isolated from water and soils, foods are considered to be the major vehicle for listeriosis [12]. Indeed, this bacterium is frequently isolated in agricultural, aquacultural and food processing environments [12], especially in a vast variety of ready-to-eat (RTE) foods (i.e. handled, processed, mixed, cooked or prepared into edible forms without further listericidal steps) [13], and may persist in food processing plants thanks to potential genes responsible for resistance to chemical compounds and biocides used for food plant sanitation [5].

Because food origins of *L.* *monocytogenes* outbreaks may be unidentified (e.g. patients who do not remember their diets the days before the first symptoms) or foodstuffs composed of food products from diverse food sectors (e.g. a salad of egg, ham and cheese) [14], and because *L.* *monocytogenes* may enter the food processing environment through employee, equipment and raw material [12], the ability to attribute efficiently (i.e. accurately and fastly) food sources to isolates is of major importance for public health authorities which track origins of foodborne outbreaks (i.e. traceback investigation) [14], and monitor *L.* *monocytogenes* in primary production, manufacturing and distribution (i.e. surveillance activity) [11] to support policy-making.

The historical source attribution models, namely STRUCTURE [15] (i.e. Bayesian clustering), modified Dutch [16] (i.e. frequency-matching) and Danish “Hald” [17] (i.e. frequency-matching) models, were mainly applied to *Campylobacter* and rarely to *Salmonella* and *Listeria*, and relied on microbial phenotypes (e.g. serotyping, phage-typing, antimicrobial resistance) or subtypes (e.g. STs, MLST, core genome MLST (cgMLST), ribosomal MLST (rMLST) and variable number tandem repeat analysis (VNTR)) [18–21]. The few studies using historical models to perform source attribution of *L.* *monocytogenes*, attributed human listerioris cases mainly to dairy products [19] or bovine reservoir [20, 21], in agreement with the unique machine learning (ML)-based study about *L. monocytogenes* source attribution (i.e. dairy products) [22].

Since the appearance of whole genome sequencing (WGS), ML models have recently been applied successfully from genomic data during the last few years to perform source attribution of pathogenic foodborne bacteria such like *L.* *monocytogenes* [22], *S.* Typhimurium [23, 24], *Campylobacter jejuni* and *coli* [25], as well as *Escherichia coli* [26]. Despite the improvements in ML-based procedures for source attribution of pathogenic foodborne bacteria, the recently developed analytical workflows still present several differences which remain to be harmonized, such as language-dependent libraries, genomic features of interest, splitting ratios between training and testing datasets, preprocessing steps, ML models and performance metrics [22–26]. Such harmonization of genomic-based ML settings and performance metrics would improve source attribution performance and allow direct comparisons of source attribution results from independent studies using common practices.

The recently developed workflows using supervised ML models for source attribution of pathogenic foodborne bacteria are based on the caret R [22–24] or scikit-learn Python [25, 26] libraries. These R caret (classification and regression training) [27] and Python scikit-learn (machine learning built over SciPy) [28] libraries are popular in data mining and predictive analytics because they provide a large range of supervised and unsupervised algorithms. Both libraries provide functions to split data, preprocess data, set models, assess model performance and perform prediction [27, 28].

These recent ML-based workflows are usually implemented from cgMLST [22, 23, 25] and less frequently from patterns of genes [24, 26] or kmers [25], because cgMLST typing is widely used in routine surveillance of foodborne pathogens and presents the advantage to harbor a constant small amount of labeled descriptors (i.e. the cgMLST loci) allowing easy inter-laboratory model exchanges [29]. While it is not the case for these recent ML-based workflows [22–26], the single nucleotide polymorphisms (SNPs) are also commonly used to build ML models for other scientific objectives [30].

Concerning the preprocessing steps before supervised ML training, these recent ML-based workflows do not perform preprocessing [24, 25], or perform a Boruta function-based reduction [23], genome wide association study (GWAS) [26], or removal of near-zero variance (NZV) descriptors [22]. The removal of NZV descriptors is largely used as a supervised ML-based preprocessing step because the exclusion of NZV descriptors from the ML model may provide benefits for models that are susceptible to this particular type of descriptors [31].

Comparing nine supervised ML models, a peculiar study assessing different input genomic features for source attribution of foodborne pathogens [25] demonstrated that the workflows presenting the highest accuracies were in this order of importance: extreme gradient boosting (XGB) [32] from cgMLST, RF [33] from cgMLST and extremely randomized trees (ERT) [34] from kmers. Compared to cgMLST and kmers input, these authors also observed lower accuracies for ML models using alleles, sequences or kmers from 7-locus MLST [25]. The other supervised ML-based studies focusing on source attribution of foodborne pathogens from genomic data estimated that the highest accuracy was reached with the boosted logistic regression (a.k.a. Logit boost: BLR) model [35] from cgMLST, compared with RF [23], or compared with RF, support vector machine (SVM) [36] and stochastic gradient boosting (SGB) [22, 37], while another study [26] reached the highest accuracy with the SVM model from genes [36] compared with Gaussian naive Bayes (GaussianNB) [38], decision trees (Dts) [39] and RF [33]. It must be noted that the SGB model [22] is an improvement of the generalized boosted model (GBM), and that the BLR [23] and multi-nomial logistic regression (MLR) [24] models are highly similar [40].

Among other possible methods for accuracy estimation of supervised ML models [41–44], the recent supervised ML-based workflows aiming at performing source attribution based on genomic data [22–26] agree to perform a similar non-exhaustive cross-validation method which does not compute all ways of splitting the original dataset of samples. More precisely, these studies combine one of the most primitive holdout method [22–26] with one of the two most advanced k-fold cross-validation: the non-repeated k-fold cross-validation [22, 23] or repeated (*n* = 10) k-fold cross-validation [24–26] methods. The holdout method aims at splitting randomly the original dataset of samples into training and testing datasets for ML model training and accuracy estimation, respectively [45]. The cross-validation method aims at splitting randomly the original dataset into k equal sized groups of samples, k-1 groups to train the model and one group to validate it, then the process is reiterated until each unique group has been used to validate the model [46]. This combined strategy allows identification and mitigation of the ML model over-fitting [47, 48]. The ML model over-fitting appears when the model trains noise (i.e. random pointless data) rather than only signal (i.e. useful data explaining the phenotype of interest), and is defined when a model matches well its training data (i.e. high accuracy and low error rate), while performing poorly in view of its validation or testing data (i.e. low accuracy and high error rate) [48]. The k-fold cross-validation is one of the most well-known methods to mitigate the over-fitting because it implies the selection of the best parameters during cross-validation and the use of these best parameters to train the retained model [47].

Concerning the holdout method [45], the proportions of training samples are usually defined higher than the proportions of testing samples by authors of the most recent articles using supervised ML for source attribution of pathogenic foodborne bacteria based on genomic data (i.e. between 70/30% and 90/10% for the training/testing datasets) [22–26]. Authors consider actually the Pareto Principle stating that roughly 80% of effects come from 20% of causes [49–52], in agreement with a recent ML-based study proposing a possible statistical reason why a splitting ratio between 70/30% and 80/20% (i.e. training/testing) provides empirically the highest prediction performance [53]. Nevertheless, the 80–20 rule may no longer be the best practice for splitting of training and testing datasets because authors confirmed [54], or not [55–57], that the optimal splitting ratio is 70/30%. As regards the k-fold cross-validation method [46], these ML based studies for source attribution of foodborne bacteria [22, 26] proposed to perform 10 [22, 26], 7 [23] or 5 [25] -fold cross-validations. While three of these four studies do not harmonize the splitting ratios between the holdout and k-fold cross-validation methods [22, 23, 25], the other one keeps equal these splitting ratios for these two methods [26]. Indeed, the larger the training dataset of the k-fold cross-validation is, the longer the ML model computational time is, and even longer for the repeated k-fold cross-validation [46].

In addition to the usual accuracy estimation (i.e. correctly classified observations both positive and negative), recent studies using supervised ML for source attribution of foodborne bacteria based on genomic data [22–26], propose to use other class-dependent performance metrics, such like precision (i.e. proportion of true positives among true and false positives) [58], recall (i.e. proportion of true positives among true positives and false negatives) [58], Cohen’s kappa (i.e. consistency across raters taking into account the agreement occurring by chance) [59] and F1-score (i.e. accuracy in terms of harmonic mean of precision and recall) [60]. One of these studies [26] propose also to use metrics from model scores to assess performance of supervised ML models through probabilistic framework of area under the curve (AUC) [61] measuring the proportion of the entire two-dimensional area underneath the entire receiver operating characteristic curve (ROC) [62], precision recall curve (PR) [63] when the datasets are highly skewed [58, 64] or precision recall gain curve (PRG) [65] in the case of both weighted and unweighted datasets [66]. It must be noted that a calibration curve (CC) can also be used to assess ML model performance, even if it does not harbor associated AUC [67, 68].

In view of these most recent articles performing ML-based source attribution of foodborne bacteria from genomic data [22–26] and with the ultimate goal to harmonize ML practices for efficient source attribution of *L.* *monocytogenes* from genomic data, we built (i) a robust collection of samples and (ii) a versatile ML workflow, in order to compare (iii) performance metrics and (iv) unstandardized ML settings. The performance metrics included accuracy [22–26], Cohen’s kappa [69], F1-score [60], AUC [61] from ROC [62], PR [63] or PRG [65], and execution time, while the unstandardized ML settings included input genomic profiles (i.e. 7-locus alleles [25], core alleles [22, 23, 25], accessory genes [24, 26], core SNPs [30] and pan kmers [25]), dataset splitting (i.e. 50, 60, 70, 80 and 90% of training dataset [52–55]), data preprocessing (i.e. with or without NZV removal [22, 31]), and learning models (i.e. BLR [35], ERT [34], RF [70], SGB [37], SVM [36] and XGB [32]).

## Results

The building of a robust collection of *L.* *monocytogenes* paired-end reads [22] (i), together with the development of a versatile ML workflow based on practices in the field of foodborne bacteria source attribution from genomic data [22–26] (ii), allowed comparison of usual performance metrics (iii) and unstandardized ML settings (iv), in order to harmonize ML practices for efficient source attribution of *L.* *monocytogenes*.

### Robust collection of samples

The quality of a previously described collection of *L.* *monocytogenes* samples [22] was assessed rigorously in order to assure authenticity and completeness of genomic profiles used as input of the developed ML workflow (Additional file 1). The corresponding procedure detailed in material and methods, retained 1 100 paired-end reads in the final collection of food samples (Additional file 2). In agreement with others studies, these confirmed *L.* *monocytogenes* samples [71] were defined by a low level of single nucleotide variants in the core single-copy ribosomal-protein genes (SNVs) [72, 73] (i.e. 0.32 ± 0.79 SNVs), a high base calling quality (i.e. 97.89 ± 1.31% of QC30) [74, 75], a high mapping coverage (i.e. 47.37 ± 5.98X and 99.3% of depth and breadth of coverage, respectively) [29], a low assembly fragmentation (i.e. 57 ± 66 contigs) [76], and an expected genome size (i.e. 2.96 ± 0.07 Mbp) [4, 8]. While significant differences (i.e. Kolmogorov–Smirnov tests) of quality metric distributions (i.e. depth, breadth, contigs and size) were observed between phenotypes considering all CCs (Additional file 3), these differences were most of the time less significant considering the most prevalent CC5 (Additional file 4). The distribution of food sources was balanced across the collection of samples, while the distribution of CCs was not uniform across food sources (Additional file 5). A small proportion of CCs/STs was slightly associated with food sources (Additional file 5) based on a Pearson’s Chi-squared test with Yates’ continuity correction (*p* = 4.00 × 10^–4^) and multiple Chi-squared tests with Bonferroni correction (i.e. 21% of CCs/STs with *p* < 4.00 × 10^–4^, impacting 41% of samples). Food sources were scattered across the tree, while CCs/STs were mostly clustered (Additional file 6). Finally, this final collection of samples allowed identification of 7 039 pan genes (i.e. 2 472 core genes > 99% of samples and 4 567 accessory genes ≤ 99% of samples), 130 663 core variants and 660 966 pan kmers.

### Versatile ML workflow

**Fig. 1:**
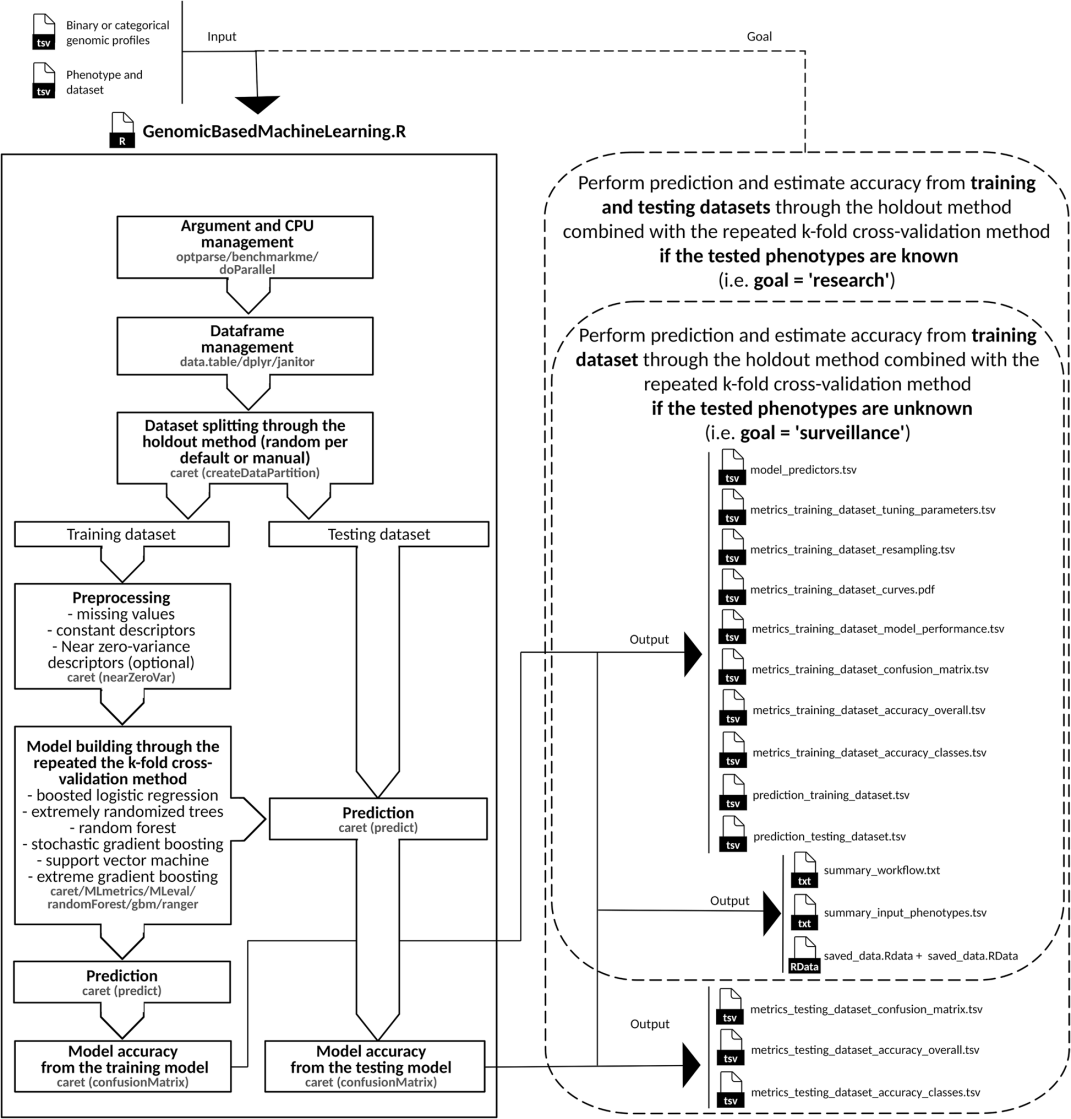
Developed workflow aiming at performing supervised machine learning for source attribution based on genomic data. The developed workflow is based on R script for research and surveillance goals and is available in Docker

The fully automatic versatile ML workflow is freely accessible in Docker for inter-laboratory exchanges and was developed according to the common and unstandardized practices (Fig. 1) described in recent studies proposing source attribution based on genomic data and ML [22–26]. In addition to be able to answer research and surveillance activities, the developed ML workflow allows modification of input genomic profiles, dataset splitting, data preprocessing, and learning models which are assessed specifically in the present study (Fig. 1). This workflow produces usual performance metrics such like accuracy [22–26], Cohen’s kappa [69], F1-score [60], AUC [61] from ROC [62], PR [63] or PRG [65], and execution time (Additional file 7). For advanced users, this versatile workflow allows also modification of other ML settings described in material and methods.

### Performance metrics

**Fig. 2:**
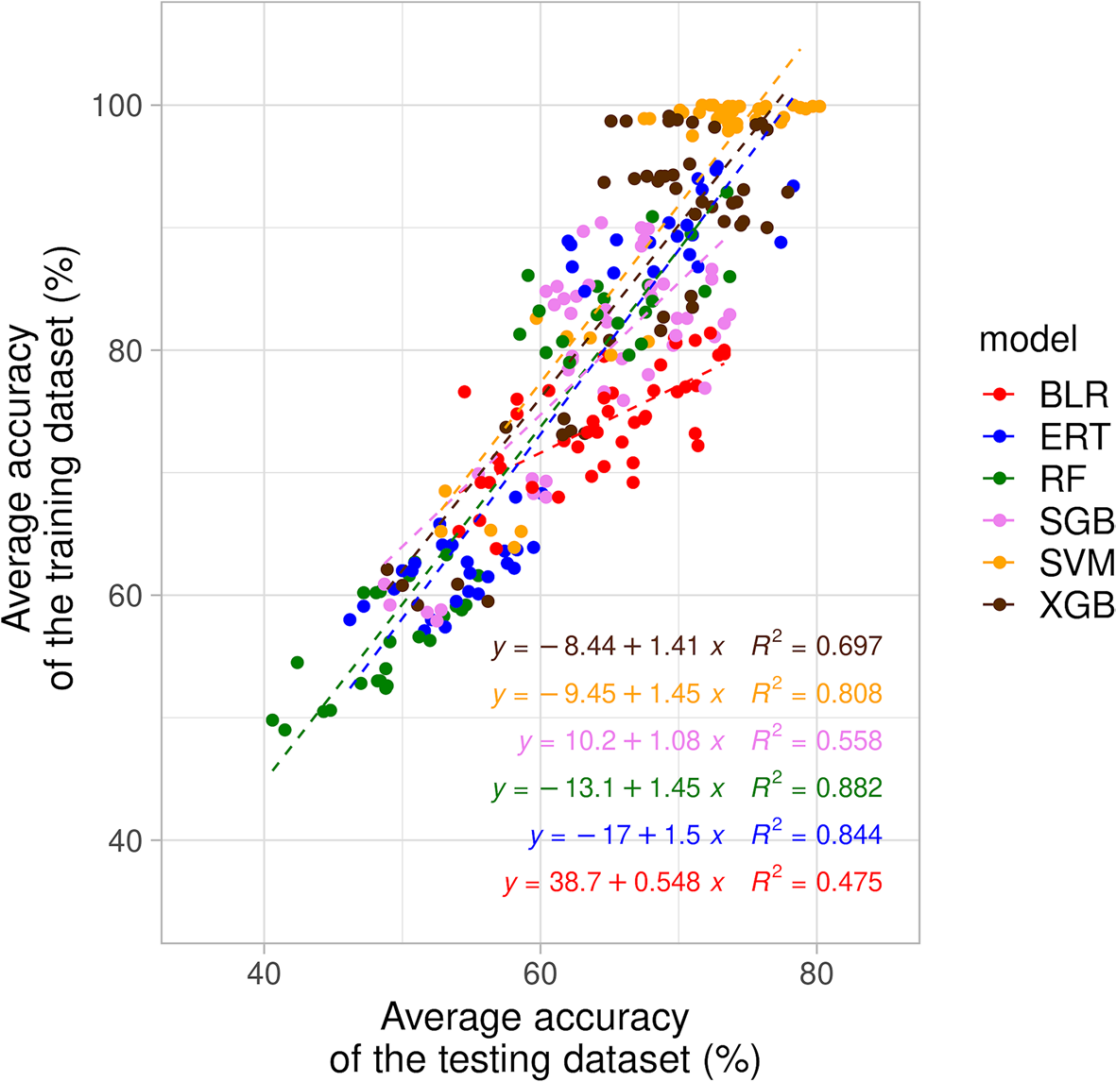
Correlations between the average accuracy of the testing dataset and average accuracy of the training dataset from different machine learning models. BLR, ERT, RF, SGB, SVM and XGB stand for boosted logistic regression, extremely randomized trees, random forest, stochastic gradient boosting, support vector machine and extreme gradient boosting, respectively

Depending on the model of interest, the average accuracy of the training dataset exhibited different levels of correlation with the average accuracy of the testing dataset (Fig. 2), as well as the other performance metrics of interest (Additional file 8A-E). The average accuracy of the testing dataset presented also different levels of correlation with the other performance metrics of interest (Additional file 8F-J). As a first noteworthy observation, the average accuracies of the training dataset were systematically higher than the corresponding average accuracies of the testing dataset (Fig. 2) and the same behavior was also observed for the other performance metrics of interest (Additional file 8). A second noticeable observation is that most of the average accuracies of the training dataset were very high and pretty constant for the SVM model (i.e. around 100%) (Fig. 2), while the corresponding average accuracies of the testing dataset were lower and more diverse (i.e. lower than around 80%) (Additional file 8). Because of these two last observations, unstandardized ML settings were compared below in view of the average accuracies of the testing dataset rather than training dataset.

### Unstandardized ML settings

**Fig. 3:**
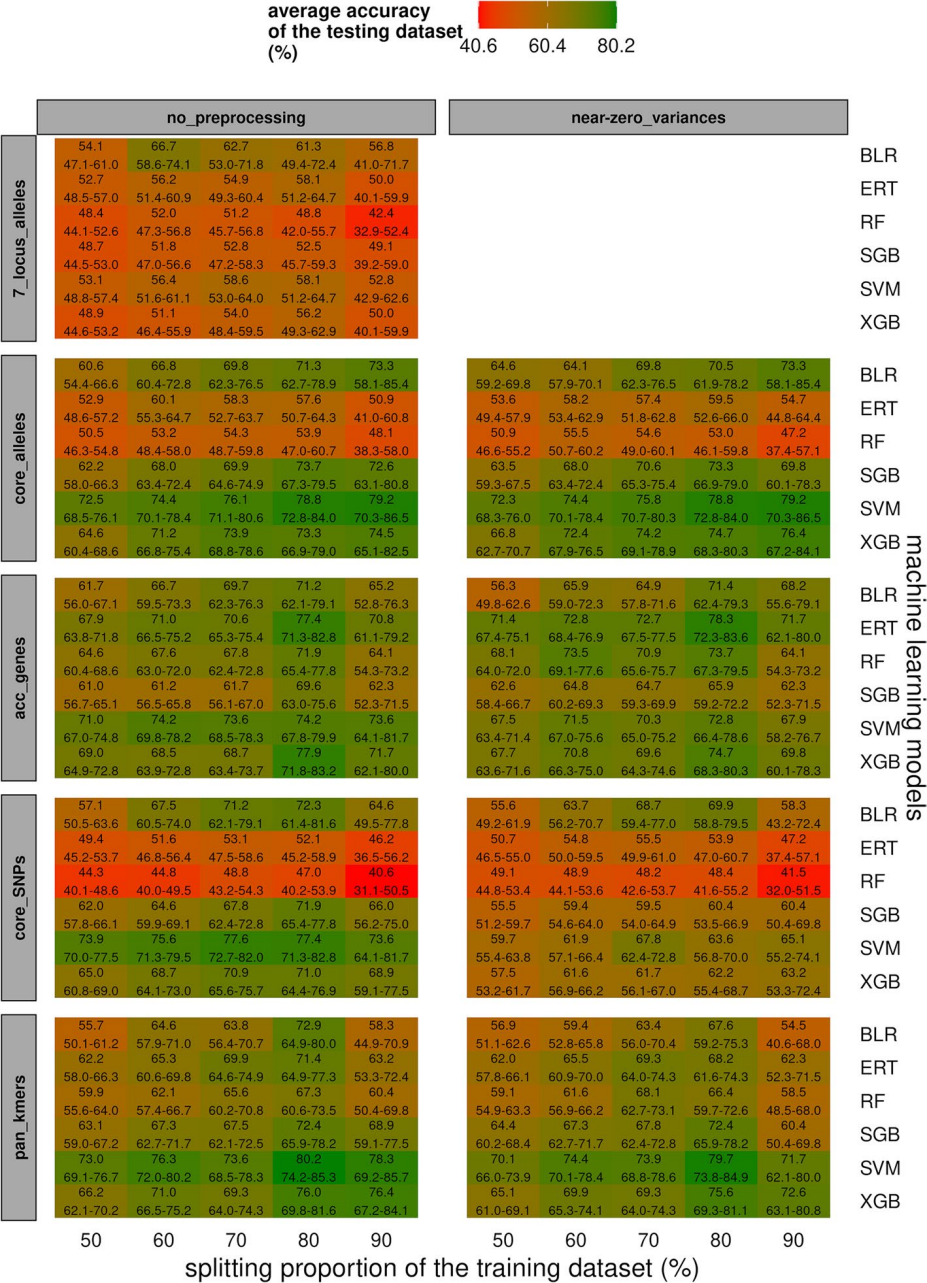
Average accuracy (i.e. top) and 95% confidence intervals (i.e. bottom) from the testing dataset (%) from different combinations of genomic profiles (i.e. 7-locus alleles, core alleles, accessory genes, core SNPs and pan kmers), dataset splitting (i.e. 50, 60, 70, 80 and 90% of training dataset), data preprocessing (i.e. with or without near-zero variance removal), and machine learning models. The splitting ratios of the holdout (50/50%, 60/40%, 70/30%, 80/20% and 90/10% for the training/testing datasets) and repeated k-fold cross-validation (k = 2.0, 2.5, 3.3, 5.0 and 10, respectively) methods were harmonized. BLR, ERT, RF, SGB, SVM and XGB stand for boosted logistic regression, extremely randomized trees, random forest, stochastic gradient boosting, support vector machine and extreme gradient boosting, respectively

Outcomes of the 7-locus alleles were not included in the analyses of variance (ANOVA) below because all corresponding performance metrics were very low (e.g. accuracy: 53.6%, CI95: 51.8–55.5) and NZV removal caused discarding of all descriptors (Additional file 9). Focusing on the accuracy from the testing dataset, ANOVA showed that the average accuracy was not impacted by the NZV removal (*p* = 0.041) and significantly impacted by genomic profiles (*p* = 2.00 × 10^–16^), ML models (*p* = 2.00 × 10^–16^) and splitting (*p* = 7.45 × 10^–10^), in this order of importance. The accuracies ranked in this order of importance: accessory genes (68.8%, CI95: 67.7–70.0), pan kmers (67.3%, CI95: 65.7–68.8), core alleles (65.7%, CI95: 63.3–68.1) and core SNPs (59.9%, CI95: 57.5–62.4). Based on Tukey multiple comparisons, the accuracy observed between pan kmers and core alleles (*p* = 0.333), or pan kmers and accessory genes (*p* = 0.353) were not significantly different, while the other pairwise comparisons of genomic features presented significant differences of accuracy (5.47 × 10^–3^ < *p* < 1.00 × 10^–8^). Interestingly, the ERT and RF models did not perform well for the core alleles and SNPs in comparison with accessory genes and pan kmers (Additional files 8 and 9). For each ML model of each genomic profile, the accuracy and training dataset splitting presented a tendency to increase gradually, reaching most of the time the highest accuracy for 80% of training dataset splitting (Additional file 9). Tukey multiple comparisons confirmed that the accuracies from 80% of training dataset splitting (67.6%, CI95: 65.1–70.1) were significantly higher that those from 50% (*p* = 1.00 × 10^–8^, 60.5%, CI95: 58.4–62.5), 60% (*p* = 6.15 × 10^–3^, 64.3%, CI95: 62.2–66.4) and 90% (*p* = 1.38 × 10^–4^, 62.8%, CI95: 59.9–65.6) of training splitting ratios. In contrast, these accuracies from 80% of training dataset splitting (67.6%, CI95: 65.1–70.1) were not significantly different than those from 70% of training dataset splitting (*p* = 0.158, 65.4%, CI95: 63.4–67.5). Even if ANOVA showed that the accuracies with (64.7%, CI95: 63.3–66.2) and without (63.6%, CI95: 62.1–65.2) NZV removal were not statistically different (*p* = 0.041), those from core SNPs appeared significantly (*p* = 3.98 × 10^–5^) lower for NZV removal (57.7%, CI95: 55.1–60.3) compared with the absence of preprocessing (62.1%, CI95: 57.9–66.4), while the NZV removal did not seem to impact accuracy for core alleles (*p* = 0.583), accessory genes (*p* = 0.982) and pan kmers (*p* = 0.020) (Fig. 3). The ML model accuracies ranked in this order of importance: SVM (71.2%, CI95: 69.0–73.3), XGB (67.8%, CI95: 65.6–70.0), BLR (64.7%, CI95: 63.0–66.4), SGB (64.0%, CI95: 62.1–65.9), ERT (60.7%, CI95: 58.1–63.3) and RF (56.4%, CI95: 53.6–59.2). Tukey multiple comparisons did not show significant differences of accuracy between the BLR and SGB (*p* = 0.999), BLR and ERT (*p* = 0.017) or SVM and XGB (*p* = 0.051), while significant differences were observed for the other pairwise investigated models (6.79 × 10^–3^ < *p* < 1.00 × 10^–8^). All these statistically supported behaviors of accuracies were also graphically confirmed based on the other assessed performance metrics (Additional file 9). Furthermore, the analyses performed with the genomic profiles harboring the highest amount of descriptors, namely core SNPs and pan kmers, were the most time-consuming, especially for the RF and SVM models and the high training dataset splittings (Additional file 9). Finally, our recommendations about ML settings presented similar phenotype class-dependent metrics for 7-locus alleles (Additional file 10A), core alleles (Additional file 10B), accessory genes (Additional file 10C), core SNPs (Additional file 10D) and pan kmers (Additional file 10E). Furthermore, additional analyses showed that class-dependent accuracies from core SNPs (i.e. 84.9%, CI95: 79.6–90.2) were not significantly different (Wilcoxon signed-rank test: *p* = 0.294) than those from only non-synonymous core SNPs (i.e. 85.9%, CI95: 80.6–91.1) based on ML settings recommended in the present study (Additional file 10).

## Discussion

A robust collection of *L.* *monocytogenes* samples (Additional files 1 and 2) [22], together with the development of a versatile ML workflow based on recently proposed ML-based methods for source attribution from genomic data (Table 1) [22–26], allowed assessment of unstandardized settings (i.e. genomic profiles [22–26, 30, 77–79], dataset splitting [52–55], data preprocessing [22, 31, 80–82] and learning models [32, 34–37, 70]) of the common holdout method [45] combined with the repeated k-fold cross-validation method [83–85], in view of usual performance metrics (i.e. accuracy [22–26], Cohen’s kappa [69], F1-score [60], AUC [61] from ROC [62], PR [63] or PRG [65], and execution time).

**Table 1:** Most recent articles using supervised machine learning for source attribution of foodborne bacteria based on genomic data. cgMSLT stands for coregenome multilocus sequence typing. ROC and AUC stand for receiver operating characteristic curve and area under the curve

**Genus (samples)**	**Input**	**Holdout method (training & testing datasets %)**	**Preprocessing**	**Cross-validation method (setting)**	**Models**	**Additional performance metrics**	**Library**	**Reference**
*Listeria* (1 366)	cgMLST	Yes (70/30)	near-zero variance	repeated k-fold cross-validation (tenfold, 10-times)	random forest + logit boost + stochastic gradient boosting + support vector machine	N/A	R caret	[22]
*Salmonella* (351)	cgMLST	Yes (70/30)	Boruta function-based reduction	repeated k-fold cross-validation (sevenfold, 10-times)	random forest + logit boost	N/A	R caret	[23]
*Salmonella* (98)	genes	Yes (70/30)	no preprocessing	cross-validation (unknown)	multinomial logistic regression	N/A	R caret	[24]
*Campylobacter* (5 799)	cgMLST + kmers	Yes (75/25)	no preprocessing	k-fold cross-validation (fivefold, 1-time)	XGBoost harboring a higher accuracy than 8 other ML models	N/A	Python scikit-learn	[25]
*Escherichia* (3 000)	genes	Yes (90/10)	GWAS	k-fold cross-validation (tenfold, 1-time)	Gaussian naive Bayes + decision trees + random forest + support vector machine	ROC curve + AUC score	Python scikit-learn	[26]

### Robust collection of samples

Special attention has been given to preparation of the collection of samples (Additional files 1 and 2) in order to include uncontaminated draft genomes (i.e. 0.32 ± 0.79) presenting high levels of Phred scores (i.e. 97.89 ± 1.31% of QC30), mapping (i.e. depth: 47.37 ± 5.98X and breadth: 92.36 ± 2.81%) and *de novo* assembly (i.e. number of contigs: 57 ± 66 and total size: 2.96 ± 0.07 Mbp) (Additional files 3 and 4). These levels of SNVs [29, 72, 86, 87], Phred scores [29, 74, 75], depth and breadth of coverage [29], number of contigs [76, 87, 88] and total size [4, 8, 87] were very similar to those described in the literature and supported authenticity and completeness of genomic profiles. In addition, the amount of core genes (i.e. 2 472 core genes) was similar to those previously described (i.e. 2 330 to 2 456 core genes [7]). Due to the higher genetic diversity assessed in the present study, the amount of accessory genes (i.e. 4 567 accessory genes) was higher than those previously described (i.e. 323 to 753 accessory genes [89]), as expected in view of our objective aiming at maximizing the amount of descriptors used to build ML models. A small proportion of CCs/STs was slightly associated with food sources (Additional files 5 and 6) in agreement with a recent study [90]. Consequently, the consideration as descriptors of CCs/STs may lightly contribute to ML performance improvement, if these MLST-derived CCs/STs are not already encoded indirectly into genomic data of interest (e.g. MLST, cgMLST, core SNPs, pan kmers and potentially accessory genes). The performance metrics (Additional files 7, 8, and 9, Figs. 2 and 3) confirmed that this collection of *L.* *monocytogenes*, presenting balanced phenotypes and non-uniform distribution of CCs across phenotypes (Additional files 5 and 6), is suitable to perform ML-based source attribution from genomic data as initially demonstrated based on cgMLST profiles by Tanui et al. [22].

### Versatile ML workflow

The developed ML workflow is versatile for several reasons. Firstly, it answers research (i.e. known phenotypes from the testing dataset) and surveillance activities (i.e. unknown phenotypes from the testing dataset) [91]. Easily installable in different operating system through the related publicly available Docker container [92], this workflow gives also the opportunity to the users to modify input genomic profiles [22–26, 30, 77–79], dataset splitting [52–55], data preprocessing [22, 31, 80–82], learning models [32, 34–37, 70], as well as settings of NZV, resampling and main tuning parameter range [27]. Compatible with binary (e.g. accessory genes and pan kmers) or categorical (e.g. 7-locus alleles, core alleles and core SNPs) genomic profiles of bacteria or virus, as well as any kind of binary (e.g. resistant or sensitive to a chemical) or multiclass (e.g. food sources) phenotypes, this ML workflow can be used in many fields such as pathogen control, genome engineering or synthetic biology [93]. The current version of the developed ML workflow does not provide ready-to-use trained models in order to give the opportunity to users to train models by themselves because successful ML analyses are often guided by the quality and quantity of descriptors [94].

### Performance metrics

The tested performance metrics were not systematically correlated (Fig. 2 and Additional file 8) because they are designed to assess specific performance elements, making them suitable for their own purposes. In addition, the fact that the performance metrics of the training dataset were systematically higher than those of the testing dataset for all considered models (Fig. 2 and Additional files 7 and 8), might signify that ML models over-fit to some extent, and/or that the collection of *L.* *monocytogenes* did not reach the optimal size representing the whole genetic diversity. An objective way to assess over-fitting only from the training model, such like the ML learning curves [95], should consequently be implemented in future versions of the developed versatile ML workflow, and this workflow should also be used to process more *L.* *monocytogenes* genomes [86].

### Unstandardized ML settings

The observation that the accuracies from accessory genes and pan kmers were significantly higher than those from core alleles and core SNPs, respectively (Fig. 3 and Additional file 9) may be explained by the fact that mutations associated to food sources are mainly observed in the accessory genome rather than the core genome, as previously demonstrated concerning the adaptation of *L.* *monocytogenes* to biocides used in food processing plants [96] or the adaptation of *Salmonella* to animal hosts [97]. According to Arning et al. [25], we also observed poor performance metrics from 7-locus MLST in comparison to core alleles, accessory genes, core SNPs and pan kmers (Fig. 3 and Additional file 9). In agreement with the Pareto Principle stating that roughly 80% of effects come from 20% of causes [52], as well as the recent ML-based study of Gholamy et al. proposing a possible statistical explanation about the optimal training dataset splitting between 70 and 80% [53], we observed independently of the considered genomic profiles and ML models that the accuracies from 70 and 80% of training dataset splitting were not significantly different, while the accuracies from 80% of training dataset splitting were significantly higher than those from 50%, 60% and 90% of training dataset splitting (Fig. 3 and Additional file 9). The NZV removal did not provide results for 7-locus alleles, did not impact significantly the accuracy for core alleles, accessory genes and pan kmers, decreased significantly accuracy for core SNPs (Fig. 3), decreased the amount of descriptors used to build models (Additional file 7), and decreased slightly the execution time (Additional file 9). As expected [31], the NZV removal appeared to be advantageous to decrease the amount of descriptors, circumvent the library-dependent limits related to long vectors and avoid negative impact on accuracy [27, 98]. Even if the SVM [36] and XGB [32] models did not present significant differences of accuracy and reached significantly higher accuracy than BLR [35], SGB [37], ERT [34] and RF [70] in this order of magnitude (Fig. 3), the SVM model popularized in the early 2000s required much more computing power than the recently popularized XGB model, especially for high amount of descriptors such like core SNPs and pan kmers (Additional file 9).

### Perspectives

In addition to recently proposed ML-based methods for source attribution from genomic data (Table 1) [22–26], other genomic data based-ML workflows for source attribution have been published during the acquisition of the present results (Fig. 3 and Additional files 7 and 9), for instance through an ultra-fast hierarchical machine learning (hML) classifier from reads’ kmers of *Salmonella* (preprint [99] and published [100]), a neighbour group classifier from 7-locus MLST profiles of *Campylobacter jejuni* (preprint [101]), and a cgMLST-based classifier from cgMLST profiles of *Legionella pneumophila* (preprint [102]). A continuous comparison of new ML workflows (i.e. performance metrics and ML settings) is consequently necessary to harmonize practices in the field of genomic data-based ML for source attribution. To face the rare unstandardized studies about *L.* *monocytogenes* source attribution making difficult to compare source attribution results from these independent studies [19–22], the performance of the ML-based source attribution workflow standardized in the present study [22–26] and historical source attribution models [19–21] should be compared in a near future. With the objective to process more genomic profiles through the developed ML workflow, we plan to investigate the intrinsic limit of the “train” function from the caret R library (version 6.0–94) [27] which does not support long vectors in contrast with other libraries [98], as well as other pre-processing steps dealing with descriptors related to bacterial population structure [103], such like collapsing of correlated descriptors [104] and removal of descriptors less relevant than random probes through the Boruta algorithm [105, 106]. As very recently proposed by other authors using genomic data to predict eae-positive Shiga toxin-producing *Escherichia coli* [107], we also plan to implement ready-to-use trained ML models into future versions of the presented ML workflow in order to speed up the real-time prediction of phenotypes for the surveillance activity [91]. Furthermore, we also plan to implement an automatic selection of the most efficient model before ML prediction as very recently proposed by other authors using genomic data to predict antimicrobial resistance of *Streptococcus pneumoniae* [106]. It would also be interesting to implement highly recognized model interpretation analyses, such like permutation variable importance [108] and/or shapley additive explanations [109], in order to assess importance of descriptors. Future comparisons of genomic data-based ML workflows with minimal multi-locus distance methodology (MMD) may also participate to the improvement of source attribution of pathogenic microorganisms [110].

## Conclusion

The present study confirmed that source attribution of *L.* *monocytogenes* can be performed through genomic data-based ML and provided recommendations about unstandardized ML settings including genomic profiles, dataset splitting, data preprocessing and learning models for the common holdout method combined with the repeated k-fold cross-validation method. More precisely, we recommend to use preferably genomic profiles from accessory or pan genomes rather than core genome, a training dataset splitting between 70 and 80%, and the XGB or SVM models, requiring modest and large computing facilities, respectively. The critical confrontation of past and newly developed ML workflows will continue to harmonize analytical procedures and provide good practices in the field of source attribution of pathogenic microorganisms for outbreak investigations and surveillance activities.

## Material and methods

A robust collection of *L.* *monocytogenes* paired-end reads presenting associated food source and the development of a versatile ML workflow fitting the research and surveillance activities, made it possible to provide recommendations about the most efficient ML workflow settings based on several performance metrics.

### Collection of paired-end reads

Based on 1 365 sample accessions (SAMN) of *L. monocytogenes* previously published in several BioProjects [22], SAMN and sequencing run accessions (SRR) were associated based on the European Nucleotide Archive (ENA) metadata, then a total of 1 421 reads available in the ENA were downloaded through wget commands. As detailed below, samples were discarded from the original collection due to filtering steps related to archive metadata, as well as metrics associated to paired-end read, reference mapping and *de novo* assembly. Concerning the archive metadata, we discarded 55 single reads and technical sequencing replicates (i.e. multiple SRR IDs for the same sample), 28 erroneous biological replicates (i.e. identical SRR IDs for multiple SAMN IDs), 3 unavailable reads in ENA and 133 clinical samples because the present study focuses on source attribution performance (i.e. food sources). With regard to the paired-end read metrics, initial reads downsampling was performed at 40X of read depth of coverage with BBNorm (version October 19, 2017) [111], as recently proposed for precise cgMLST typing of *L.* *monocytogenes* [29]. From this collection of 1 202 samples, 16 paired-end reads were discarded because they were contaminated by exogenous DNA (i.e. lower than 10 single nucleotide variants (SNVs) [72]), according to ConFindr (version 0.7.4) outcomes [73]. After a control of the expected species with Kraken (version 1.0) [71], 13 additional paired-and reads were discarded because they harbored less than 90% Phred scores higher than QC30 (quality control), according to FastQC (version 0.11.5) outcomes [112] and in agreement with a previous assessment of parameters required for precise cgMLST typing [29]. In view of mapping metrics, 32 samples with depth of coverage values below 35X and 27 samples with breadth of coverage values below 85% were rejected. The read depth of coverage was measured with BBmap (February 13, 2020) [111] against the *L.* *monocytogenes* EGD-e reference genome (i.e. NC_003210.1) [113]. Concerning the assembly metrics, 14 samples with more than 400 total contigs were discarded and the genome sizes were controlled (i.e. < 3.5 Mbp). The final collection of samples was constituted of 1 100 paired-end reads (Additional file 1).

### *De novo* assembly

As previously described [29], genome assembly was performed from downsampled paired-end reads with the in-house NGSmanager *de novo* assembly pipeline from the GENPAT information system implemented in IZSAM. Briefly, the NGSmanager assembly pipeline performed read trimming with Trimmomatic (version 0.36; clipping 2:30:10; leading 25; trailing 25 sliding window 20:25 minimal length 36) [114], *de novo* assembly with SPAdes (version 3.11.1; only assembler; careful; -k 21, 33, 55 and 77) [115], and filtering of contigs lower than 200 bp with a custom Python script AssemblyFilter.py (i.e. version 2.7.8). The assembly quality was assessed with Quast (version 4.4) [116] and the assembly annotation was performed with Prokka (version 1.14.5) [117]. Independently of the NGSmanager *de novo* assembly pipeline, the assembly metrics were compiled through the Bourne-Again shell (a.k.a. bash) interpreter [118].

### Genomic features of interest

The present study aims at evaluating suitable settings of ML workflow according to usual input genomic features including 7-locus alleles, core alleles, accessory genes, core SNPs and pan kmers. The accessory genes and pan kmers were encoded through binary profiles, while the 7-locus alleles, as well as core alleles and SNPs, were encoded through categorical profiles. The format of these genomic features were harmonized through the bash interpreter [118] in agreement with the usual tsv format (i.e. genomic features in lines and samples in columns [119, 120]).

#### 7-locus alleles

CCs and STs were identified from draft assemblies with MLST (version 2.16.1) [121]. The 7-locus alleles were retrieved and compiled through the bash interpreter [118]. The novel full length allele similar to a “n” known allele (i.e. encoded “ ~ n”) and partial match to a “n” known allele (i.e. encoded “n?”) were considered as non-determined (ND) alleles in the present study.

#### Core alleles

cgMLST allele profiles were identified from draft assemblies with chewBBACA (version 2.6.0) [122] as described by the cross-sectoral platform for the integration of genomics in the surveillance of foodborne pathogens (INNUENDO) [123]. More precisely, default settings of chewBBACA (including allele size threshold = 0.2, BLASTP score ratio ≥ 0.6 and the recommended prodigal training file Listeria_monocytogenes.trn: https://chewbbaca.online/stats [124]) were applied in the present study. The exact matches with alleles from the schema (encoded EXC), new inferred allele (INF), locus not found (LNF), possible locus on the tip of contigs (PLOT), alleles larger (ALM) and smaller (ASM) than mode were considered in the present study, while non-informative paralogous hits (NIPH) and non-informative paralogous exact match (NIPHEM) were considered as missing data. The profiles of cgMLST alleles were identified based on the *L.* *monocytogenes* schema of 1 748 cgMLST loci [125] downloaded from BIGSdb-Lm [125, 126] as recently described [29]. The cgMLST format was transformed and transposed into the expected input ML format (i.e. genomic features in lines and samples in columns) with the bash interpreter [118].

#### Accessory genes

The pangenomic genes including core and accessory genes were extracted from annotation output of draft assemblies with Panaroo (version 1.2.3) [119] through strict mode (i.e. genes present in at least 5% of genomes), excluding invalid genes (i.e. premature stop codons), using default threshold defining homologous genes (i.e. 95% of sequence identity, 70% of protein family sequence identity, and 98% of length difference) and merging paralogous genes.

#### Core SNPs

Also implemented into our GENPAT information system, variants including SNPs and small insertions/deletions (InDels) were identified from paired-end reads (i.e. downsampled and trimmed) with the Snippy pipeline (version 4.5.1) [127] presenting a strong and uniform performance across species [128] based on BWA-based mapping [129] and FreeBayes-based variant calling [130]. The Snippy pipeline (i.e. single-sample dependent vcf files) performed mapping, variant calling and variant annotation against the annotated *L.* *monocytogenes* EGD-e reference genome (i.e. NC_003210.1) [113]. The Snippy-core pipeline (i.e. multi-samples dependent vcf file) retained only core SNPs [127]. The vcf format was transformed and transposed into the expected input ML format (i.e. genomic features in lines and samples in columns) with the bash interpreter [118].

#### Pan kmers

The kmtricks program was used from paired-end reads (i.e. downsampled and trimmed) to generate presence/absence profiles of kmers representing non-erroneous kmers from each paired-end reads (i.e. low abundance) and avoiding rare kmers, useless in a ML context (i.e. low recurrence) [131]. Firstly, the kmtricks module named “pipeline” produced partitions of kmers based on kmer size of 100 bases (i.e. “kmer-size” argument), low abundance of 10 kmers per single sample (i.e. “hard-min” argument), low recurrence of 20 kmers across samples (i.e. “recurrence-min” argument), random sub-selection of 5% (i.e. “restrict-to” argument), matrix mode of kmer presence/absence profiles into bin output (i.e. “kmer:pa:bin” string of the “mode” argument) and compression of temporary files (i.e. “cpr” argument). Secondly, the kmtricks module named “aggregate” aggregated partitions of kmers based on presence/absence profiles of kmers (i.e. “kmer” string of the “pa-matrix” argument) and generated human readable format of kmer profiles (i.e. “text” string of the “format” argument) from compressed inputs (i.e. “cpr-in” argument). The sample identifiers were added to the dataframe of presence/absence profiles of accessory kmers with the bash interpreter [118] following the order of sample identifiers provided by the kmtricks input file [131].

### Machine learning workflow

The “GenomicBasedMachineLearning.R” workflow (version 1.0) was developed to fit requirements of *L.* *monocytogenes* source attribution for research or surveillance goals described below (Fig. 1). Due the intrinsic limit of the “train” function from the caret R library (version 6.0–94) [27] which does not support long vectors yet [98], randomly selections of SNPs and kmers were performed through the bash interpreter (i.e. the “shuf” function) [118] and this limit has been estimated around 46 thousand descriptors for the present study.

#### Mandatory goals and input files

For reasons of usage simplicity, the developed ML workflow requires a goal, an input file encoding binary (e.g. accessory genes and pan kmers) or categorical (e.g. 7-locus alleles, core alleles and core SNPs) genomic profiles (i.e. “mutations” argument) in tab-separated values format (i.e. Roary [120] or Panaroo-like [119] output file with genomic features in lines and samples in columns), and an input tsv file encoding phenotypes (i.e. “phenotype” argument). More precisely, the goals fit research or surveillance activities, because the workflow can estimate training model accuracy through holdout [45] and repeated k-fold cross-validation [46] methods and perform prediction if testing phenotypes are known (i.e. “research” string of the “goal” argument), or if testing phenotypes are unknown (i.e. “surveillance” string of the “goal” argument). The double check of accuracy (i.e. from the training and testing datasets) allows to set properly the ML workflow during research activity, and then perform phenotype prediction of unknown tested samples with a single step of accuracy checking during surveillance activity (i.e. from the training dataset). Furthermore, we decided to use a tsv file of input genomic profiles because it fits well the usual encoding of variants, genes and kmers (i.e. genotype in rows and samples in columns), and also because it is simple to derive a vcf file of core SNPs [132] into a tsv file of alternative variant profiles through the most popular interpreter bash [118].

#### Data management

The workflow was developed with the R language (version 4.3.0) [133] and RStudio integrated development environment (version 2022.02.3, build 492) [134] through the Ubuntu 20.04.5 LTS (Focal Fossa) distribution. The versions of R libraries were controlled with the remote R library (version 2.4.2). The container image of this R-based workflow was built from a Rocker image managing the R version (https://rocker-project.org/images/versioned/r-ver.html) through the Docker platform (version 20.10.22, build 3a2c30b) [92]. The workflow arguments were managed with the optparse R library inspired by Python optparse library (version 1.7.3). The available central processing unit (CPUs) and parallel job processing were managed by default with the benchmarkme (version 1.0.8) and doParallel (version 1.0.17) [135–137] R libraries, respectively. It must be noted that the libssl-dev and libcurl4-openssl-dev libraries were installed into the Ubuntu 20.04.5 LTS (Focal Fossa) distribution in order to install properly the benchmarkme (version 1.0.8) R library. The user can also specify the number of CPUs to use (i.e. “cpu” argument). The reading and manipulation of dataframes were performed with the data.table (version 1.14.8) and dplyr (version 1.1.2) R libraries. The descriptors containing potential missing data (encoded as absence of string) were discarded systematically with the base R library (version 4.3.0) [133]. The descriptors harboring potential constant values (e.g. core genes from Panaroo [119]) were discarded with the janitor R library (version 2.2.0).

#### Holdout method

According to the Pareto Principle (a.k.a. the 80–20 rule [49]) and results of the present study, we implemented per default 80/20% (i.e. training/testing datasets) of randomized and stratified splitting through the “createDataPartition” function of the caret R library (version 6.0–94) [27] (i.e. “random” string of the “dataset” argument). Because this optimal ratio of the holdout method may be dependent on the selected model [56] and dataset size [57], we implemented an optional argument into the ML workflow to control this dataset splitting (i.e. “splitting” argument) in order to allow testing of different splitting ratios (i.e. training/testing datasets). If necessary during research activity aiming at defining stability of prediction performance according to controlled datasets, the user can also define himself the training and testing samples into the mandatory tsv input file dedicated to phenotypes and dataset labeling (i.e. “manual” string of the “dataset” argument).

#### Descriptor preprocessing

The highly used method removing NZV descriptors [22, 31] was implemented into the developed ML workflow (i.e. “variances” argument) from training samples and through the “nearZeroVar” function of the caret R library (version 6.0–94) [27]. This “nearZeroVar” function of the caret R library allows removal of NZV descriptors presenting a high fraction of unique values over the sample size (i.e. “uniqueCut” argument) and a low ratio between the frequency of the most prevalent value and the frequency of the second most prevalent value (i.e. “freqCut” argument) [27]. The present study used the default values of the “uniqueCut” (i.e. 10) and “freqCut” (i.e. 19) arguments of the “nearZeroVar” function and we decided to implement these two thresholds as arguments of the developed ML workflow because users may wish to modify the “uniqueCut” (i.e. “unique” argument) and “freqCut” (i.e. “ratio” argument) arguments due to the fact that the number of NZV descriptors depend on the dataset size [31]. Because one of our main objectives was to perform ML from binary (e.g. accessory genes and pan kmers) or categorical (e.g. 7-locus alleles, core alleles and core SNPs) genomic profiles, we decided to consider all these descriptors as categorical variables and refrain from implementing typical preprocessing steps intended for numerical descriptors, namely removal of highly correlated descriptors and descriptor transformations (e.g. centering, scaling, BoxCox, YeoJohnson, exponential and principal component analysis) with the “findCorrelation” and “preProcess” functions of the caret R library, respectively [27].

#### Non-exhaustive cross-validation method

According to the recent supervised ML-based workflows aiming at performing source attribution based on genomic data [22–26], the holdout method [45] combined with repeated k-fold cross-validation method [46] were implemented into the workflow developed in the present study. According to the Pareto Principle (a.k.a. the 80–20 rule [49]) and results of the present study, the fivefold cross-validation method was implemented per default into the developed workflow through the “trainControl” function of the caret R library (version 6.0–94) [27]. We also implemented an optional argument into the ML workflow to control the k-fold cross-validation (i.e. “fold” argument) in order to allow testing of different k [22–26]. According to Im et al. (2022) about the judicious adjustment of splittings from the holdout and repeated k-fold cross-validation methods [26], the present study tested several splitting ratios harmonizing those from the holdout (50/50%, 60/40%, 70/30%, 80/20% and 90/10% for the training/testing datasets) [45] and repeated k-fold cross-validation (k = 2.0, 2.5, 3.3, 5.0 and 10, respectively) methods [46]. According to Tanui et al*.* (2022) [22] and Munck et al*.* (2020) [23] about the advantage to perform repeated k-fold cross-validations, the developed ML workflow implement 10 repetitions per default of the k-fold cross-validation method [22, 23]. In case of user needs, the number of repetitions was also implemented as an argument of the developed ML workflow (i.e. “repetition” argument).

#### ML models

The developed ML workflow implements the ML models (i.e. “fit” argument) presenting the highest performances among ML models tested by the most recent studies aiming at performing source attribution of foodborne bacteria based on genomic data [22–26], namely BLR [22, 23] (similar to MLR [24]), ERT [25], RF [25], SGB [22], SVM [26] and XGB [25]. These ML models were implemented into the workflow through an optional argument (XGB per default) based on the “train” function of the caret R library (version 6.0–94) [27]. The setting of this “train” function implied the ROC metric from the MLmetrics R library (version 1.1.1). While the SVM [36] and BLR [35] models were implemented through the caret R library (version 6.0–94) [27], the RF [70], SGB [37], ERT [34] and XGB [32] models were implemented through the randomForest (version 4.7–1.1), gbm (version 2.1.8.1), ranger (version 0.15.1) [138] and xgboost (version 1.7.5.1) R libraries, respectively. Indeed, the deprecated extraTrees R library (2022–06-14) was not used in the present study to implement the ERT model [34], because check problems were not corrected by the authors despite reminders from the comprehensive R archive network (CRAN). For each of the implemented model, the “expand.grid” function of the caret R library (version 6.0–94) [27] was used in the present study based on main tuning parameters from 1 to 10 by 1 by default (i.e. arguments “nIter” for BLR, “mtry” for ERT, “mtry” for RF, “interaction.depth” for SGB, “cost” for SVM and “nrounds” for XGB). To allow the user to change this range of main tuning parameters, we implemented as argument (i.e. “tuning” argument) the maximal value of the main parameter to consider for the model tuning (i.e. default 10). More precisely, ten incremental tenth of the maximal value of the main parameter will be considered for the model tuning. Concerning the ERT model [34], this “expand.grid” function implied also the extratrees splitting rule (i.e. “splitrule” argument) and 1 minimal node size for classification (i.e. “min.node.size” argument). Concerning the SGB model [37], this “expand.grid” function implied also 20 gradient boosting iterations (i.e. “n.trees” argument), a learning rate of 0.01 (i.e. “shrinkage” argument) and 3 observations in each terminal node (i.e. “n.minobsinnode” argument). Concerning the XGB model [32], this “expand.grid” function implied also 0.3 learning rate (i.e. “eta” argument), 6 depth of the tree (i.e. “max_depth” argument), 0 regularization preventing overfitting (i.e. “gamma” argument), 1 observation supplied to a tree (i.e. “subsample” argument), 1 minimal instance required in a child node (i.e. “min_child_weight” argument) and 1 variable supplied to a tree (i.e. “colsample_bytree” argument).

#### Performance metrics

The developed ML workflow estimated global accuracy from the training and testing datasets based on the holdout method [45] combined with the repeated k-fold cross-validation method [46]. The accuracy and confidence intervals (95% CI), as well as Cohen’s kappa statistic [59] were estimated based on the “confusionMatrix” function of the caret R library (version 6.0–94) [27]. The “confusionMatrix” function of the caret R library (version 6.0–94) [27] was also used to calculate phenotype class-dependent performance metrics such like sensitivity, specificity, precision, recall and others. In addition to the accuracy estimation, other global performance metrics were implemented into the developed ML workflow. From the ML model training outcomes, the “evalm” function of the MLeval R (version 0.3) library was used in order to estimate performance metrics, such as F1-score [60], CC [68], as well as AUC [61] from ROC [62], PR [63], and PRG [65].

#### Output files

The names of output files are controlled by a prefix argument (i.e. “prefix” argument). Depending of the goal selected by the user (i.e. “goal” argument), the output files include (Fig. 1): predictors selected by the model (i.e. predictors.tsv), model fitting metrics through parameters (i.e. tuning_parameters.tsv) and resampling (i.e. resampling.tsv), performance curves (i.e. ROC, CC, PR, PRG: curves.pdf) and performance metrics (i.e. accuracy, Cohen’s kappa, F1score, ROC-AUC, PR-AUC, PR-AUC and others: performance.tsv), confusion matrix (i.e. confusion_matrix.tsv), overall accuracy (i.e. accuracy_overall.tsv), accuracy per phenotype of interest (i.e. accuracy_classes.tsv), prediction (i.e. prediction.tsv), and workflow summary (i.e. summary_workflow.txt). In order to reuse the same dataset splitting for new run, the output files includes also an input phenotype summary (i.e. summary_input_phenotypes.tsv). Finally, an external representation of R objects (i.e. saved_data.RData) and a short-cut of the current workspace (i.e. saved_images.RData) can be produced for downstream developments (i.e. “backup” argument, default FALSE).

### Phylogenomic tree, statistical analyses and graphical representations

As recently proposed, the phylogenomic tree was inferred from hamming distances [139] derived from cgMLST profiles [122]. Additional available Rscripts were developed to perform non-parametric tests, graphical representations and ANOVA of the present study (i.e. boxplots.R, heatmappe.R and ANOVA.R). Mapping and assembly metrics (Additional files 3 and 4) were displayed and compared based on the ggplot2 (version 3.4.1) [140], dplyr (version 1.1.0), ape (version 5.7.1), ggprism (version 1.0.4), reshape2 (version 1.4.4) and stats (version 3.6.2) [133] R libraries, while ML performance metrics (Fig. 2 and Additional file 7) were displayed based one ggplot2 (version 3.4.1) [140], plyr (version 1.8.8), ggpmisc (version 0.5.2), reshape2 (version 1.4.4) and lubridate (version 1.9.2) [141] R libraries. The Pearson’s Chi-squared test with Yates’ continuity correction and multiple Chi-squared tests with Bonferroni correction were performed based on the stats (version 4.3.1) [133] and pacman (version 0.5.1) R libraries, respectively. Concerning the ANOVA assessing the impacts of unstandardized ML settings on accuracy, the dataframes were managed with the R library dplyr (version 1.1.0), the homogeneity of variances was confirmed with Levene’s tests from the car R library (version 3.1.2) for the variables splitting (*p* = 0.121) and preprocessing (*p* = 0.826), and the Normal distribution of ANOVA residues was confirmed with Shapiro–Wilk tests from the stats R library (version 3.6.2) [133] for all genomic profiles together (*p* = 0.196) and each of them independently (0.045 < *p* < 0.588). Multi-way ANOVA was performed with the “aov” function of the ggpubr R library (version 0.6.0) assuming homogeneity of variances and Normal distribution of ANOVA residues, while one-way ANOVA was performed with the “oneway.test” function of the stats R library (version 3.6.2) [133] assuming heterogeneity of variances and Normal distribution of ANOVA residues. Tukey multiple pairwise-comparisons of means were performed with the “TukeyHSD” function of stats R library (version 3.6.2) [133]. Concordant results between these multi-way and one-way ANOVA were provided in the present study.

### Supplementary Information

**Additional file 1.** Filtration steps aiming at preparing the collection of *Listeria monocytogenes* genomes for machine learning-based source attribution.

**Additional file 2.** Filtrated collection of *Listeria monocytogenes* genomes used in the present machine learning‑based study for source attribution.

**Additional file 3.** Boxplot-based distributions of depth of coverage (A), breadth of coverage (B), number of contigs (C) and total genome length (D) from the filtrated collection of *Listeria monocytogenes* paired-end reads.

**Additional file 4.** Boxplot-based distributions of depth of coverage (A), breadth of coverage (B), number of contigs (C) and total genome length (D) from the filtrated collection of *Listeria monocytogenes* paired-end reads across the most frequent clonal complex 5.

**Additional file 5.** Distribution of clonal complexes and *p*-value of association tests.

**Additional file 6.** Phylogenomic tree inferred from hamming distances derived from cgMLST profiles.

**Additional file 7.** Metrics of machine learning performance from different combinations of genomic profiles (i.e. 7‑locus alleles, core alleles, accessory genes, core SNPs and pan kmers), dataset splitting (i.e. 50, 60, 70, 80 and 90% of training dataset), data preprocessing (i.e. with or without near‑zero variance removal), and machine learning models.

**Additional file 8.** Correlations between the average accuracy of the training (A-E) or testing (F-G) datasets and Cohen’s kappa (A and F), F1-score (B and G), as well as area under the curve (AUC) from the receiver operating characteristic (ROC) (C and H), precision recall (PR) (D and I) or precision recall gain (PRG) (E and J) curves from different machine learning models.

**Additional file 9.** Cohen’s kappa from the testing dataset (A), F1-score (B), as well as area under the curve (AUC) from the receiver operating characteristic (ROC) (C), precision recall (PR) (D) or precision recall gain (PRG) (E) curves, and execution time (F) from different combinations of genomic profiles (i.e. 7-locus alleles, core alleles, accessory genes, core SNPs and pan kmers), dataset splitting (i.e. 50, 60, 70, 80 and 90% of training dataset), data preprocessing (i.e. with or without near-zero variance removal), and machine learning models.

**Additional file 10.** Phenotype class‑dependent metrics estimated from the testing dataset with ML settings recommended in the present study (i.e. 80% of training dataset splitting, no near‑zero variance removal and SVM model) from 7‑locus alleles (A), core alleles (B), accessory genes (C), core SNPs (D) and pan kmers (E).

## Data Availability

The paired-end reads are available in the ENA under the BioProjects described in supplementary data (Additional file 1). The source code of graphical representations, non-parametric tests and ANOVA are available in GitHub (https://github.com/PCas95/GenomicBasedMachineLearning). The source code of the machine learning workflow is available in GitHub (https://github.com/Nicolas-Radomski/GenomicBasedMachineLearning) and Docker (https://hub.docker.com/r/nicolasradomski/genomicbasedmachinelearning).

## Abbreviations

ALM: Alleles larger than mode
ANOVA: Analysis of variance
ASM: Alleles smaller than mode
AUC: Area under the curve
bash: Bourne-Again shell
BLR: Boosted logistic regression
caret: Classification and regression training
CC: Calibration curve
CCs: Clonal complexes
cgMLST: Coregenome multi-locus sequence typing
CI: Confidence intervals
CPUs: Central processing unit
CRAN: Comprehensive R archive network
ENA: European nucleotide archive
ERT: Extremely randomized trees
EU: European Union
EXC: Exact matches
GBM: Generalized boosted model
GENPAT: Whole Genome Sequencing of microbial pathogens: data-base and bioinformatics analysis
hML: Hierarchical machine learning
InDels: Small insertions/deletions
INF: New inferred allele
IZSAM: Istituto Zooprofilattico Sperimentale dell’Abruzzo e del Molise “Giuseppe Caporale”
LNF: Locus not found
LOOCV: Leave one out cross validation
MLST: Multi-locus sequence typing
MMD: Multi-locus distance methodology
MLR: Multi-nomial logistic regression
ND: Non-determined
NIPH: Non-informative paralogous hits
NRC: National Reference Centre
NZV: Near-zero variance
PLOT: Possible locus on the tip of contigs
PR: Precision recall curve
PRG: Precision recall gain curve
RF: Random forest
rMLST: Ribosomal multi-locus sequence typing
ROC: Receiver operating characteristic curve
RTE: Ready-to-eat
SAM: Sample accession
scikit-learn: Machine learning built over SciPy
SGB: Stochastic gradient boosting
SNVs: Single nucleotide variants
STs: Sequence types
SRR: Sequencing run accession
SVM: Support vector machine
VNTR: Variable number tandem repeat analysis
WGS: Whole genome sequencing
XGB: Extreme gradient boosting
