# Supplementary material for: Harmonization of supervised machine learning practices for efficient source attribution of Listeria monocytogenes based on genomic data
Source: BMC Genomics. 2023 Sep 22;24:560. doi: 10.1186/s12864-023-09667-w (PMC10515079; doi:10.1186/s12864-023-09667-w)

**Additional file 1: Filtration steps aiming at preparing the collection of *Listeria monocytogenes* genomes for machine learning-based source attribution.** The reference article corresponds to Tanui *et al*. (2022, Pathogens; 11(6):691). Data was retrieved from ENA (European Nucleotide Archive). Exogenous DNA contamination was detected with Confindr. Species were identified with Kraken. Depth and breadth of coverage were estimated with BBmap and the *L. monocytogenes* EGD-e reference genome (i.e. NC_003210.1). Total length and number of contigs were identified with Quast.

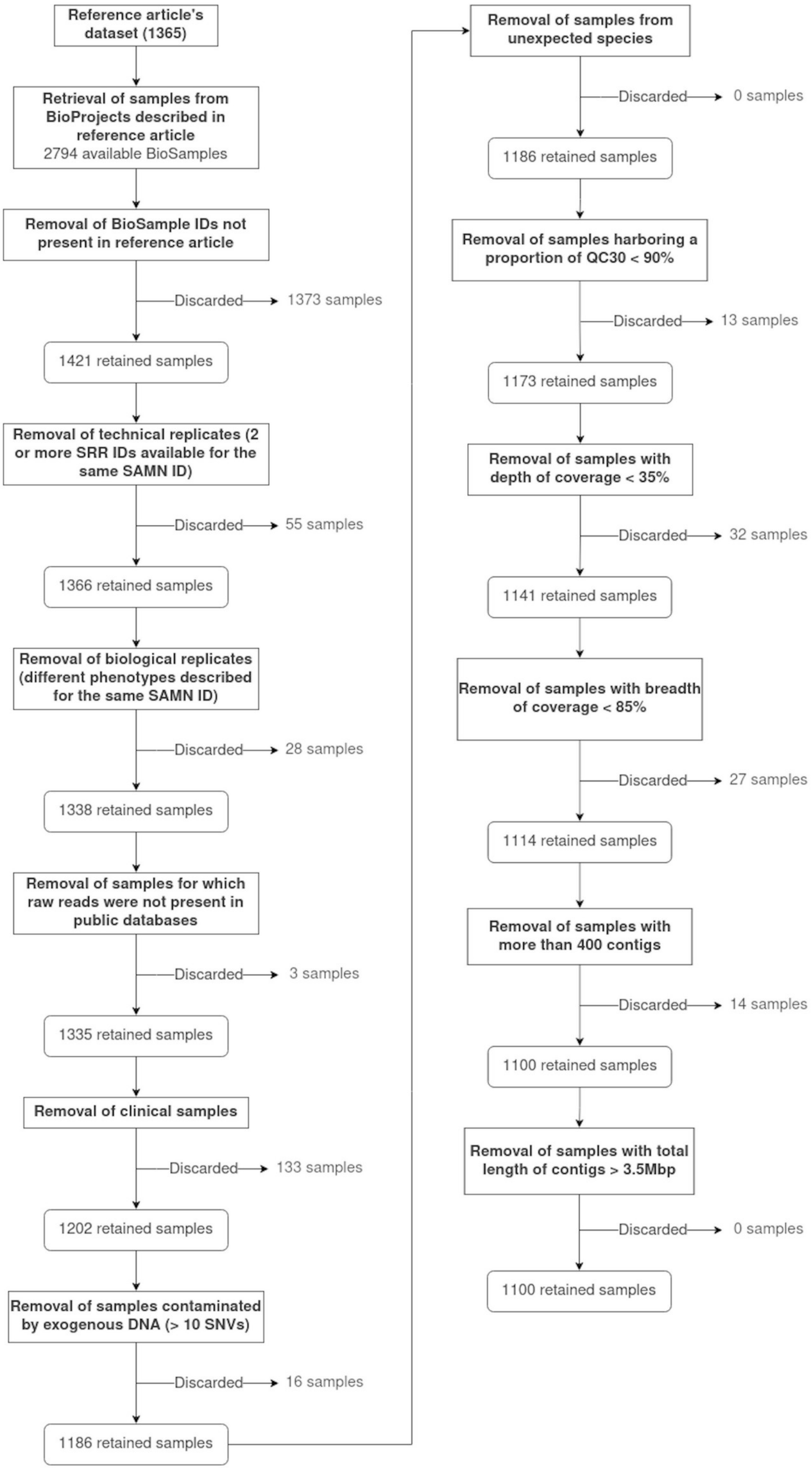

Supplement: Supplementary file 1 — Additional file 1. Filtration steps aiming at preparing the collection of Listeria monocytogenes genomes for machine learning-based source attribution. [file 12864_2023_9667_MOESM1_ESM.pdf]
