# Supplementary material for: Harmonization of supervised machine learning practices for efficient source attribution of Listeria monocytogenes based on genomic data
Source: BMC Genomics. 2023 Sep 22;24:560. doi: 10.1186/s12864-023-09667-w (PMC10515079; doi:10.1186/s12864-023-09667-w)

Additional file 3: Boxplot-based distributions of depth of coverage (A), breadth of coverage (B), number of contigs (C) and total genome length (D) from the filtrated collection of *Listeria monocytogenes* paired-end reads. Breadth and depth of coverage were estimated with BBmap. Number of contigs and total length were compiled with Quast. These distributions across phenotypes were compared with non-parametric Kolmogorov-Smirnov tests.

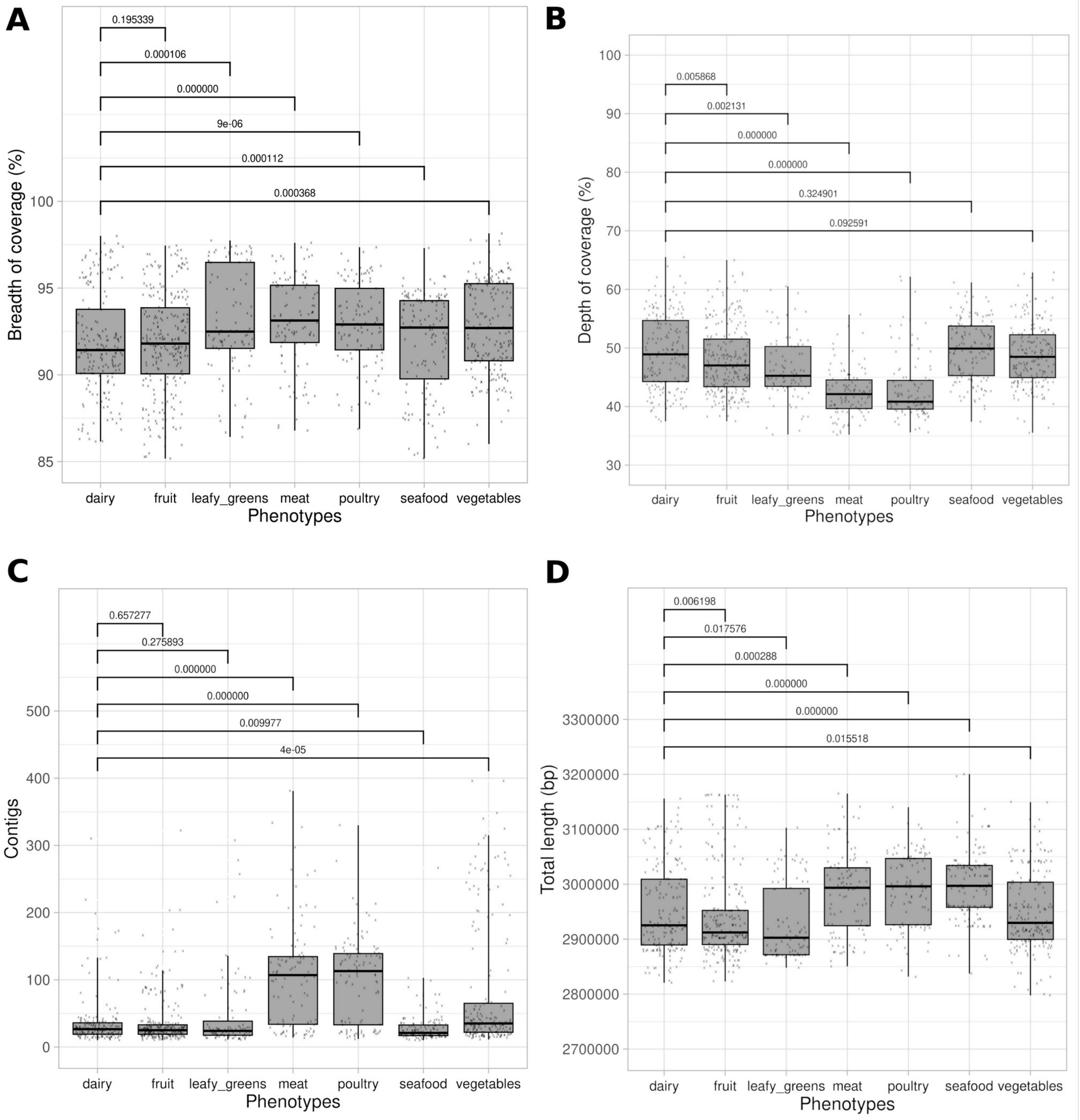

Supplement: Supplementary file 3 — Additional file 3. Boxplot-based distributions of depth of coverage (A), breadth of coverage (B), number of contigs (C) and total genome length (D) from the filtrated collection of Listeria monocytogenes paired-end reads. [file 12864_2023_9667_MOESM3_ESM.pdf]
