# Supplementary material for: Harmonization of supervised machine learning practices for efficient source attribution of Listeria monocytogenes based on genomic data
Source: BMC Genomics. 2023 Sep 22;24:560. doi: 10.1186/s12864-023-09667-w (PMC10515079; doi:10.1186/s12864-023-09667-w)

Additional file 4: Boxplot-based distributions of depth of coverage (A), breadth of coverage (B), number of contigs (C) and total genome length (D) from the filtrated collection of *Listeria monocytogenes* paired-end reads across the most frequent clonal complex 5. Breadth and depth of coverage were estimated with BBmap. Number of contigs and total length were compiled with Quast. These distributions across phenotypes were compared with non-parametric Wilcoxon signed-rank tests. The clonal complexes were identified with MLST.

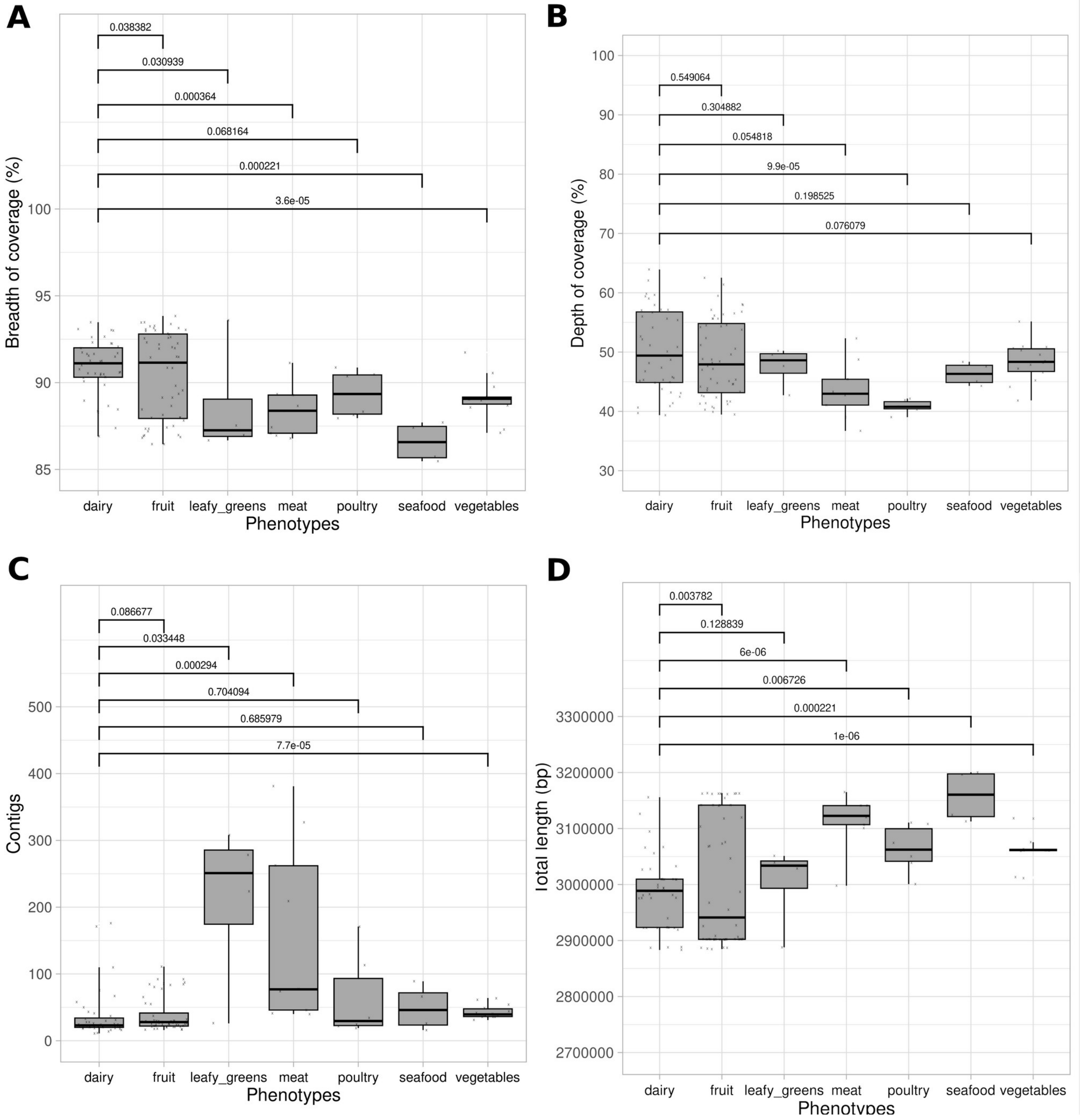

Supplement: Supplementary file 4 — Additional file 4. Boxplot-based distributions of depth of coverage (A), breadth of coverage (B), number of contigs (C) and total genome length (D) from the filtrated collection of Listeria monocytogenes paired-end reads across the most frequent clonal complex 5. [file 12864_2023_9667_MOESM4_ESM.pdf]
