# Supplementary material for: Harmonization of supervised machine learning practices for efficient source attribution of Listeria monocytogenes based on genomic data
Source: BMC Genomics. 2023 Sep 22;24:560. doi: 10.1186/s12864-023-09667-w (PMC10515079; doi:10.1186/s12864-023-09667-w)
Supplement: Supplementary file 8 — Additional file 8. Correlations between the average accuracy of the training (A-E) or testing (F-G) datasets and Cohen’s kappa (A and F), F1-score (B and G), as well as area under the curve (AUC) from the receiver operating characteristic (ROC) (C and H), precision recall (PR) (D and I) or precision recall gain (PRG) (E and J) curves from different machine learning models. [file 12864_2023_9667_MOESM8_ESM.pdf]

Additional file 8: Correlations between the average accuracy of the training (A-E) or testing (F-G) datasets and Cohen's kappa (A and F), F1-score (B and G), as well as area under the curve (AUC) from the receiver operating characteristic (ROC) (C and H), precision recall (PR) (D and I) or precision recall gain (PRG) (E and J) curves from different machine learning models. The F1-score corresponds to the F-score, also called F-measure. BLR, ERT, RF, SGB, SVM and XGB stand for boosted logistic regression, extremely randomized trees, random forest, stochastic gradient boosting, support vector machine and extreme gradient boosting, respectively.

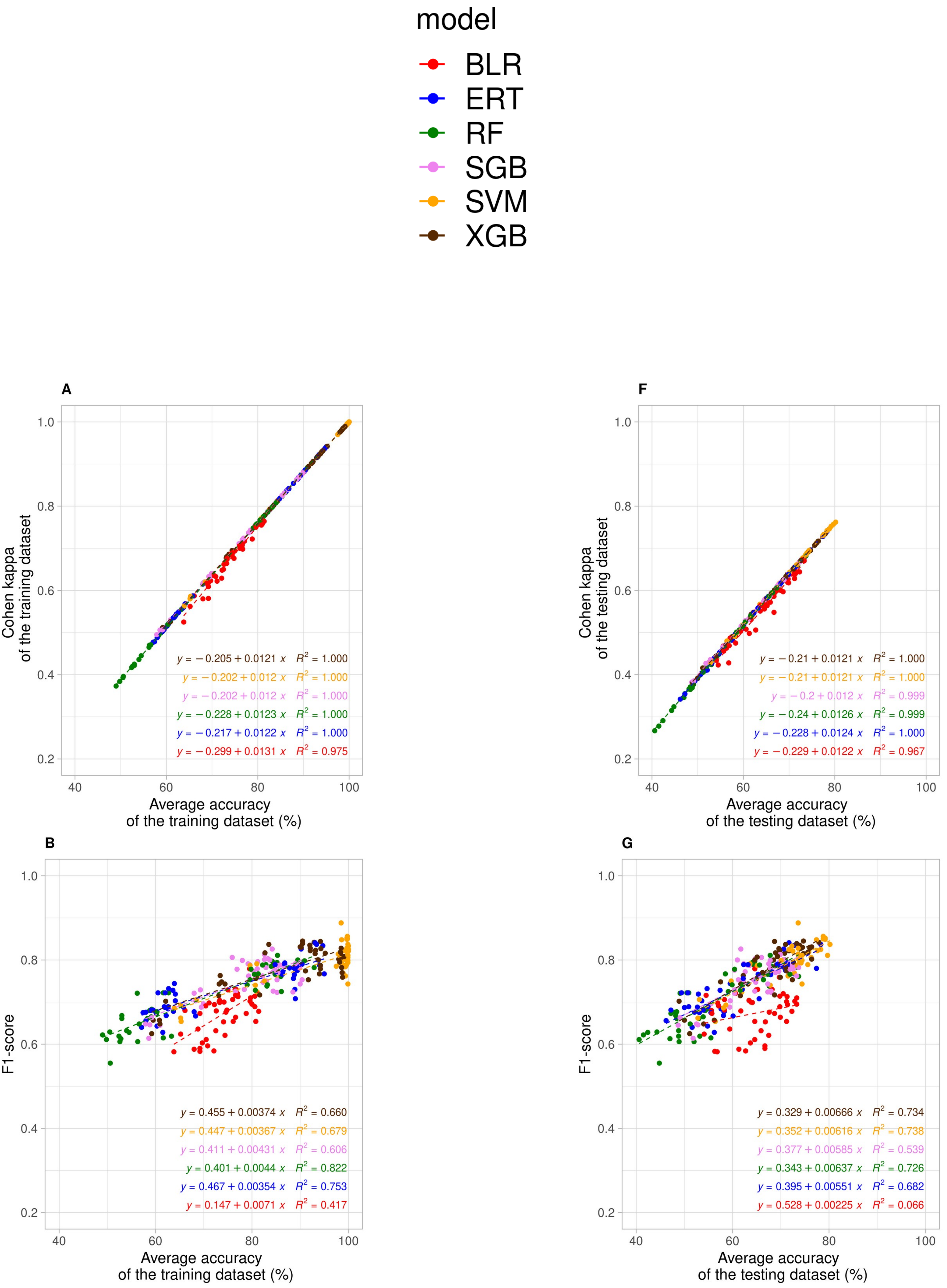
model
BLR
ERT
RF
SGB
SVM
XGB
A
Cohen kappa of the training dataset
Average accuracy of the training dataset (%)
y = −0.205 + 0.0121 x   R² = 1.000
y = −0.202 + 0.012 x   R² = 1.000
y = −0.202 + 0.012 x   R² = 1.000
y = −0.228 + 0.0123 x   R² = 1.000
y = −0.217 + 0.0122 x   R² = 1.000
y = −0.299 + 0.0131 x   R² = 0.975
F
Cohen kappa of the testing dataset
Average accuracy of the testing dataset (%)
y = −0.21 + 0.0121 x   R² = 1.000
y = −0.21 + 0.0121 x   R² = 1.000
y = −0.2 + 0.012 x   R² = 0.999
y = −0.24 + 0.0126 x   R² = 0.999
y = −0.228 + 0.0124 x   R² = 1.000
y = −0.229 + 0.0122 x   R² = 0.967
B
F1-score
Average accuracy of the training dataset (%)
y = 0.455 + 0.00374 x   R² = 0.660
y = 0.447 + 0.00367 x   R² = 0.679
y = 0.411 + 0.00431 x   R² = 0.606
y = 0.401 + 0.0044 x   R² = 0.822
y = 0.467 + 0.00354 x   R² = 0.753
y = 0.147 + 0.0071 x   R² = 0.417
G
F1-score
Average accuracy of the testing dataset (%)
y = 0.329 + 0.00666 x   R² = 0.734
y = 0.352 + 0.00616 x   R² = 0.738
y = 0.377 + 0.00585 x   R² = 0.539
y = 0.343 + 0.00637 x   R² = 0.726
y = 0.395 + 0.00551 x   R² = 0.682
y = 0.528 + 0.00225 x   R² = 0.066

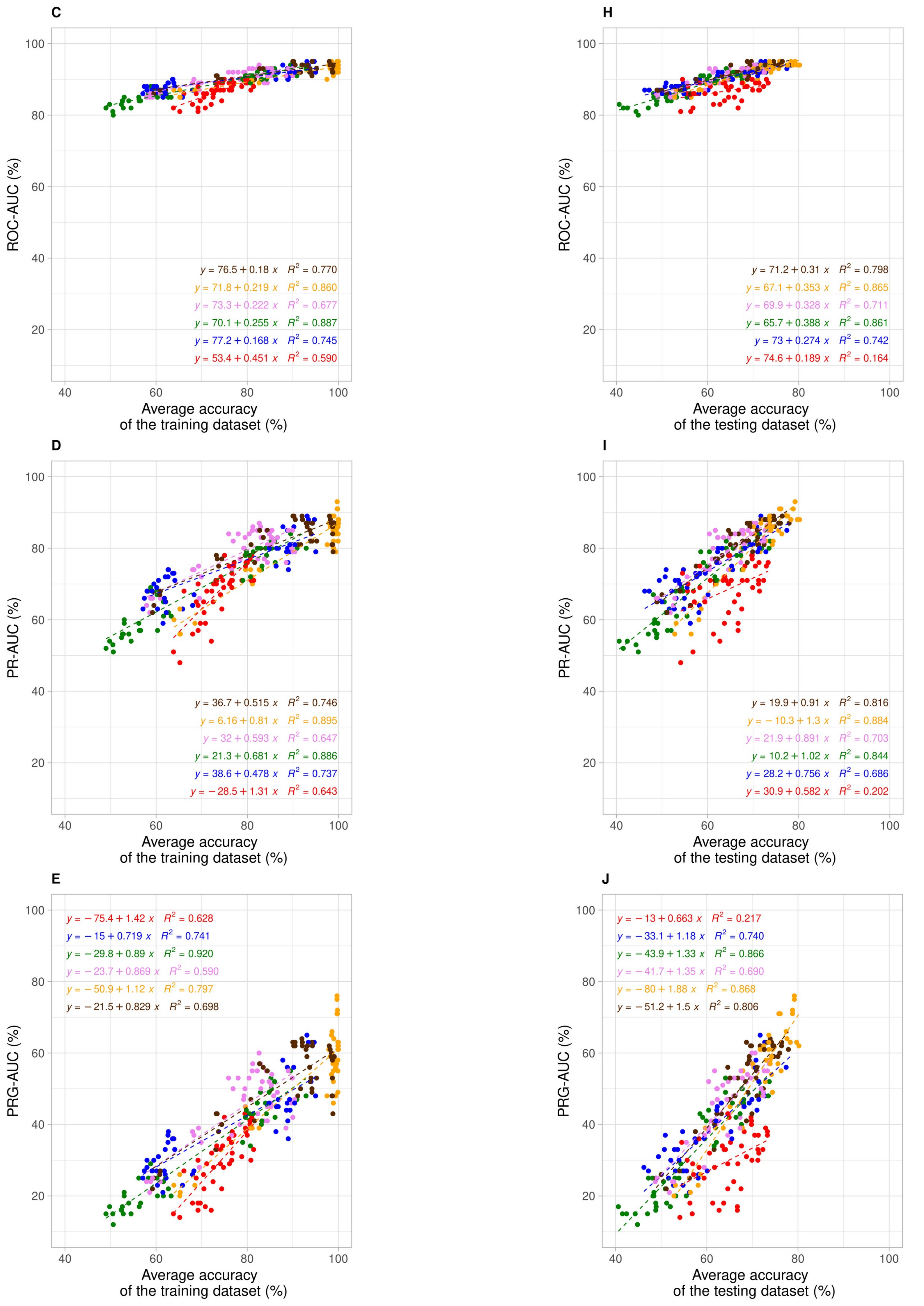

C
ROC-AUC (%)
y = 76.5 + 0.18 x R^2 = 0.770
y = 71.8 + 0.219 x R^2 = 0.860
y = 73.3 + 0.222 x R^2 = 0.677
y = 70.1 + 0.255 x R^2 = 0.887
y = 77.2 + 0.168 x R^2 = 0.745
y = 53.4 + 0.451 x R^2 = 0.590
Average accuracy of the training dataset (%)
H
ROC-AUC (%)
y = 71.2 + 0.31 x R^2 = 0.798
y = 67.1 + 0.353 x R^2 = 0.865
y = 69.9 + 0.328 x R^2 = 0.711
y = 65.7 + 0.388 x R^2 = 0.861
y = 73 + 0.274 x R^2 = 0.742
y = 74.6 + 0.189 x R^2 = 0.164
Average accuracy of the testing dataset (%)
D
PR-AUC (%)
y = 36.7 + 0.515 x R^2 = 0.746
y = 6.16 + 0.81 x R^2 = 0.895
y = 32 + 0.593 x R^2 = 0.647
y = 21.3 + 0.681 x R^2 = 0.886
y = 38.6 + 0.478 x R^2 = 0.737
y = − 28.5 + 1.31 x R^2 = 0.643
Average accuracy of the training dataset (%)
I
PR-AUC (%)
y = 19.9 + 0.91 x R^2 = 0.816
y = − 10.3 + 1.3 x R^2 = 0.884
y = 21.9 + 0.891 x R^2 = 0.703
y = 10.2 + 1.02 x R^2 = 0.844
y = 28.2 + 0.756 x R^2 = 0.686
y = 30.9 + 0.582 x R^2 = 0.202
Average accuracy of the testing dataset (%)
E
PRG-AUC (%)
y = − 75.4 + 1.42 x R^2 = 0.628
y = − 15 + 0.719 x R^2 = 0.741
y = − 29.8 + 0.89 x R^2 = 0.920
y = − 23.7 + 0.869 x R^2 = 0.590
y = − 50.9 + 1.12 x R^2 = 0.797
y = − 21.5 + 0.829 x R^2 = 0.698
Average accuracy of the training dataset (%)
J
PRG-AUC (%)
y = − 13 + 0.663 x R^2 = 0.217
y = − 33.1 + 1.18 x R^2 = 0.740
y = − 43.9 + 1.33 x R^2 = 0.866
y = − 41.7 + 1.35 x R^2 = 0.690
y = − 80 + 1.88 x R^2 = 0.868
y = − 51.2 + 1.5 x R^2 = 0.806
Average accuracy of the testing dataset (%)
