# Supplementary material for: Harmonization of supervised machine learning practices for efficient source attribution of Listeria monocytogenes based on genomic data
Source: BMC Genomics. 2023 Sep 22;24:560. doi: 10.1186/s12864-023-09667-w (PMC10515079; doi:10.1186/s12864-023-09667-w)
Supplement: Supplementary file 9 — Additional file 9. Cohen’s kappa from the testing dataset (A), F1-score (B), as well as area under the curve (AUC) from the receiver operating characteristic (ROC) (C), precision recall (PR) (D) or precision recall gain (PRG) (E) curves, and execution time (F) from different combinations of genomic profiles (i.e. 7-locus alleles, core alleles, accessory genes, core SNPs and pan kmers), dataset splitting (i.e. 50, 60, 70, 80 and 90% of training dataset), data preprocessing (i.e. with or without near-zero variance removal), and machine learning models. [file 12864_2023_9667_MOESM9_ESM.pdf]

Additional file 9: Cohen’s kappa from the testing dataset (A), F1-score (B), as well as area under the curve (AUC) from the receiver operating characteristic (ROC) (C), precision recall (PR) (D) or precision recall gain (PRG) (E) curves, and execution time (F) from different combinations of genomic profiles (i.e. 7-locus alleles, core alleles, accessory genes, core SNPs and pan kmers), dataset splitting (i.e. 50, 60, 70, 80 and 90% of training dataset), data preprocessing (i.e. with or without near-zero variance removal), and machine learning models. The splitting ratios of the holdout (50/50%, 60/40%, 70/30%, 80/20% and 90/10% for the training/testing datasets) and repeated k-fold cross-validation (k = 2.0, 2.5, 3.3, 5.0 and 10, respectively) methods were harmonized. The F1-score corresponds to the F-score, also called F-measure. BLR, ERT, RF, SGB, SVM and XGB stand for boosted logistic regression, extremely randomized trees, random forest, stochastic gradient boosting, support vector machine and extreme gradient boosting, respectively. The grey cells represent extreme values that we decided to exclude from the color range of heatmap in order to increase the color contrast between the intermediate values. Execution times of the present study were estimated using 120 CPUs and 900 GB memory from a x86_64 architecture (AlmaLinux distribution release 8.6: Sky Tiger).

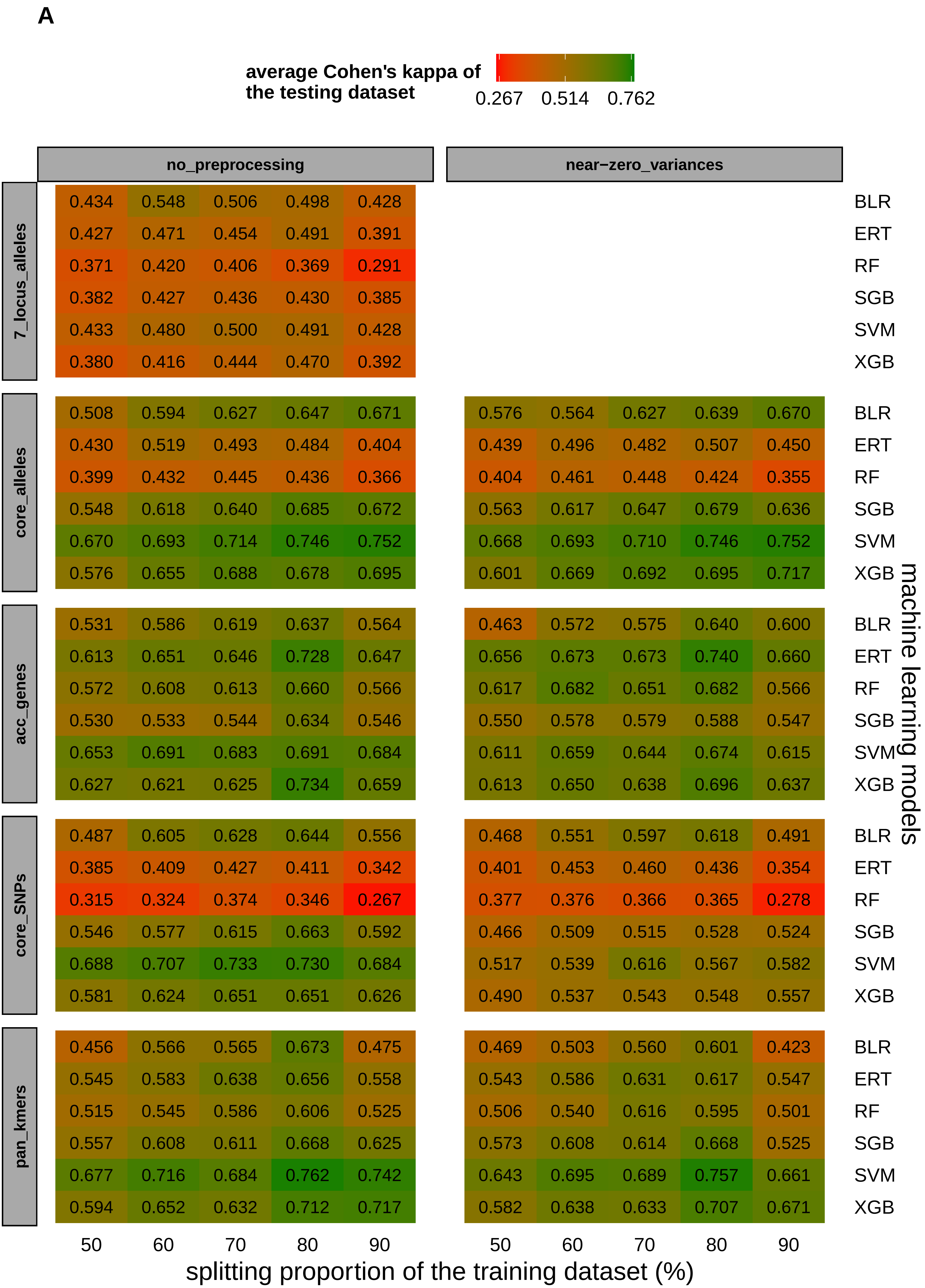
A
average Cohen's kappa of the testing dataset
0.267 0.514 0.762
no_preprocessing
near−zero_variances
7_locus_alleles
0.434 0.548 0.506 0.498 0.428 BLR
0.427 0.471 0.454 0.491 0.391 ERT
0.371 0.420 0.406 0.369 0.291 RF
0.382 0.427 0.436 0.430 0.385 SGB
0.433 0.480 0.500 0.491 0.428 SVM
0.380 0.416 0.444 0.470 0.392 XGB
core_alleles
0.508 0.594 0.627 0.647 0.671 0.576 0.564 0.627 0.639 0.670 BLR
0.430 0.519 0.493 0.484 0.404 0.439 0.496 0.482 0.507 0.450 ERT
0.399 0.432 0.445 0.436 0.366 0.404 0.461 0.448 0.424 0.355 RF
0.548 0.618 0.640 0.685 0.672 0.563 0.617 0.647 0.679 0.636 SGB
0.670 0.693 0.714 0.746 0.752 0.668 0.693 0.710 0.746 0.752 SVM
0.576 0.655 0.688 0.678 0.695 0.601 0.669 0.692 0.695 0.717 XGB
acc_genes
0.531 0.586 0.619 0.637 0.564 0.463 0.572 0.575 0.640 0.600 BLR
0.613 0.651 0.646 0.728 0.647 0.656 0.673 0.673 0.740 0.660 ERT
0.572 0.608 0.613 0.660 0.566 0.617 0.682 0.651 0.682 0.566 RF
0.530 0.533 0.544 0.634 0.546 0.550 0.578 0.579 0.588 0.547 SGB
0.653 0.691 0.683 0.691 0.684 0.611 0.659 0.644 0.674 0.615 SVM
0.627 0.621 0.625 0.734 0.659 0.613 0.650 0.638 0.696 0.637 XGB
core_SNPs
0.487 0.605 0.628 0.644 0.556 0.468 0.551 0.597 0.618 0.491 BLR
0.385 0.409 0.427 0.411 0.342 0.401 0.453 0.460 0.436 0.354 ERT
0.315 0.324 0.374 0.346 0.267 0.377 0.376 0.366 0.365 0.278 RF
0.546 0.577 0.615 0.663 0.592 0.466 0.509 0.515 0.528 0.524 SGB
0.688 0.707 0.733 0.730 0.684 0.517 0.539 0.616 0.567 0.582 SVM
0.581 0.624 0.651 0.651 0.626 0.490 0.537 0.543 0.548 0.557 XGB
pan_kmers
0.456 0.566 0.565 0.673 0.475 0.469 0.503 0.560 0.601 0.423 BLR
0.545 0.583 0.638 0.656 0.558 0.543 0.586 0.631 0.617 0.547 ERT
0.515 0.545 0.586 0.606 0.525 0.506 0.540 0.616 0.595 0.501 RF
0.557 0.608 0.611 0.668 0.625 0.573 0.608 0.614 0.668 0.525 SGB
0.677 0.716 0.684 0.762 0.742 0.643 0.695 0.689 0.757 0.661 SVM
0.594 0.652 0.632 0.712 0.717 0.582 0.638 0.633 0.707 0.671 XGB
50 60 70 80 90 50 60 70 80 90
splitting proportion of the training dataset (%)
machine learning models

B

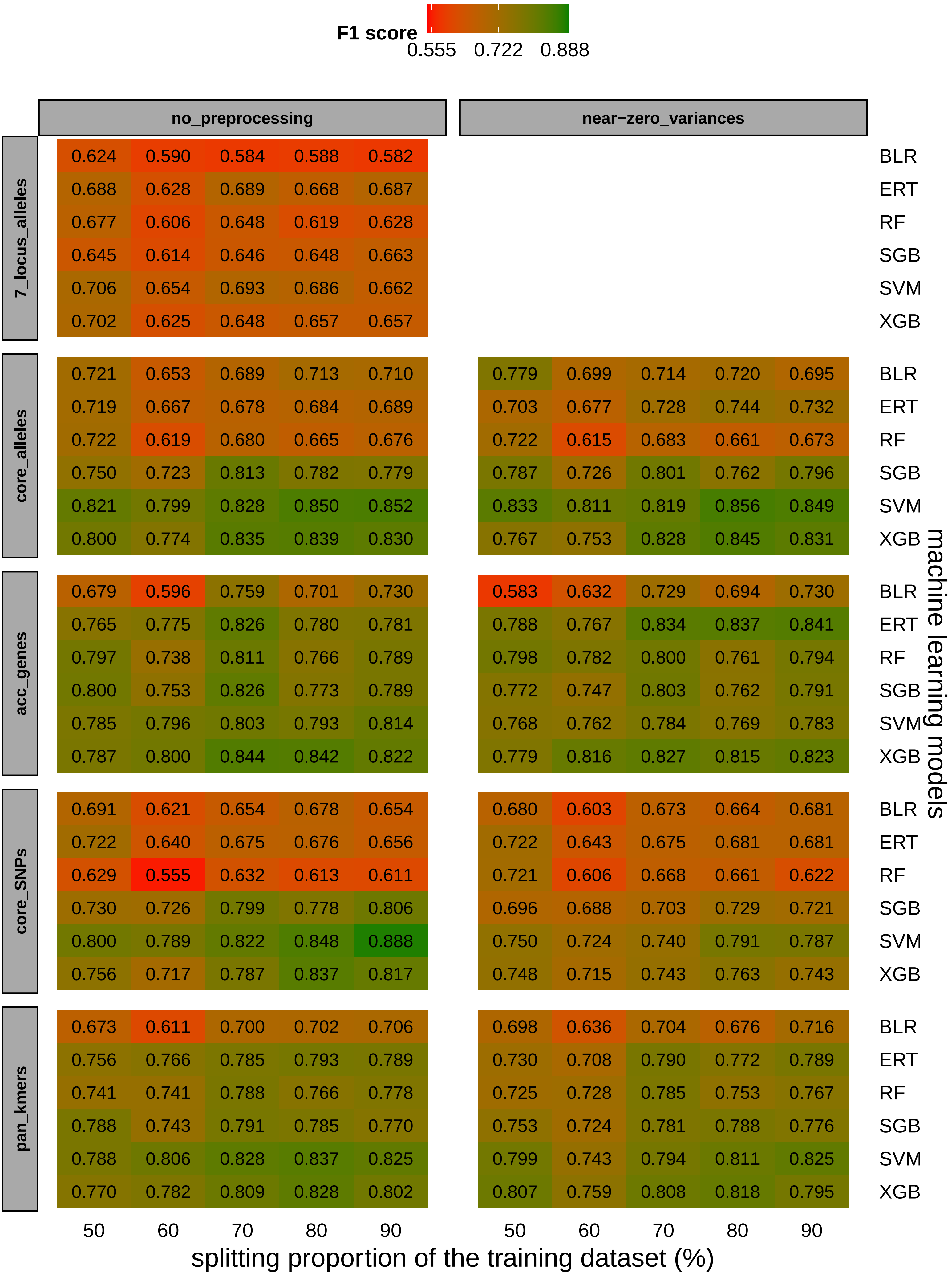
F1 score
0.555 0.722 0.888
no_preprocessing
near−zero_variances
7_locus_alleles
BLR 0.624 0.590 0.584 0.588 0.582
ERT 0.688 0.628 0.689 0.668 0.687
RF 0.677 0.606 0.648 0.619 0.628
SGB 0.645 0.614 0.646 0.648 0.663
SVM 0.706 0.654 0.693 0.686 0.662
XGB 0.702 0.625 0.648 0.657 0.657
core_alleles
BLR 0.721 0.653 0.689 0.713 0.710 | 0.779 0.699 0.714 0.720 0.695
ERT 0.719 0.667 0.678 0.684 0.689 | 0.703 0.677 0.728 0.744 0.732
RF 0.722 0.619 0.680 0.665 0.676 | 0.722 0.615 0.683 0.661 0.673
SGB 0.750 0.723 0.813 0.782 0.779 | 0.787 0.726 0.801 0.762 0.796
SVM 0.821 0.799 0.828 0.850 0.852 | 0.833 0.811 0.819 0.856 0.849
XGB 0.800 0.774 0.835 0.839 0.830 | 0.767 0.753 0.828 0.845 0.831
acc_genes
BLR 0.679 0.596 0.759 0.701 0.730 | 0.583 0.632 0.729 0.694 0.730
ERT 0.765 0.775 0.826 0.780 0.781 | 0.788 0.767 0.834 0.837 0.841
RF 0.797 0.738 0.811 0.766 0.789 | 0.798 0.782 0.800 0.761 0.794
SGB 0.800 0.753 0.826 0.773 0.789 | 0.772 0.747 0.803 0.762 0.791
SVM 0.785 0.796 0.803 0.793 0.814 | 0.768 0.762 0.784 0.769 0.783
XGB 0.787 0.800 0.844 0.842 0.822 | 0.779 0.816 0.827 0.815 0.823
core_SNPs
BLR 0.691 0.621 0.654 0.678 0.654 | 0.680 0.603 0.673 0.664 0.681
ERT 0.722 0.640 0.675 0.676 0.656 | 0.722 0.643 0.675 0.681 0.681
RF 0.629 0.555 0.632 0.613 0.611 | 0.721 0.606 0.668 0.661 0.622
SGB 0.730 0.726 0.799 0.778 0.806 | 0.696 0.688 0.703 0.729 0.721
SVM 0.800 0.789 0.822 0.848 0.888 | 0.750 0.724 0.740 0.791 0.787
XGB 0.756 0.717 0.787 0.837 0.817 | 0.748 0.715 0.743 0.763 0.743
pan_kmers
BLR 0.673 0.611 0.700 0.702 0.706 | 0.698 0.636 0.704 0.676 0.716
ERT 0.756 0.766 0.785 0.793 0.789 | 0.730 0.708 0.790 0.772 0.789
RF 0.741 0.741 0.788 0.766 0.778 | 0.725 0.728 0.785 0.753 0.767
SGB 0.788 0.743 0.791 0.785 0.770 | 0.753 0.724 0.781 0.788 0.776
SVM 0.788 0.806 0.828 0.837 0.825 | 0.799 0.743 0.794 0.811 0.825
XGB 0.770 0.782 0.809 0.828 0.802 | 0.807 0.759 0.808 0.818 0.795
50 60 70 80 90 | 50 60 70 80 90
splitting proportion of the training dataset (%)
machine learning models

C

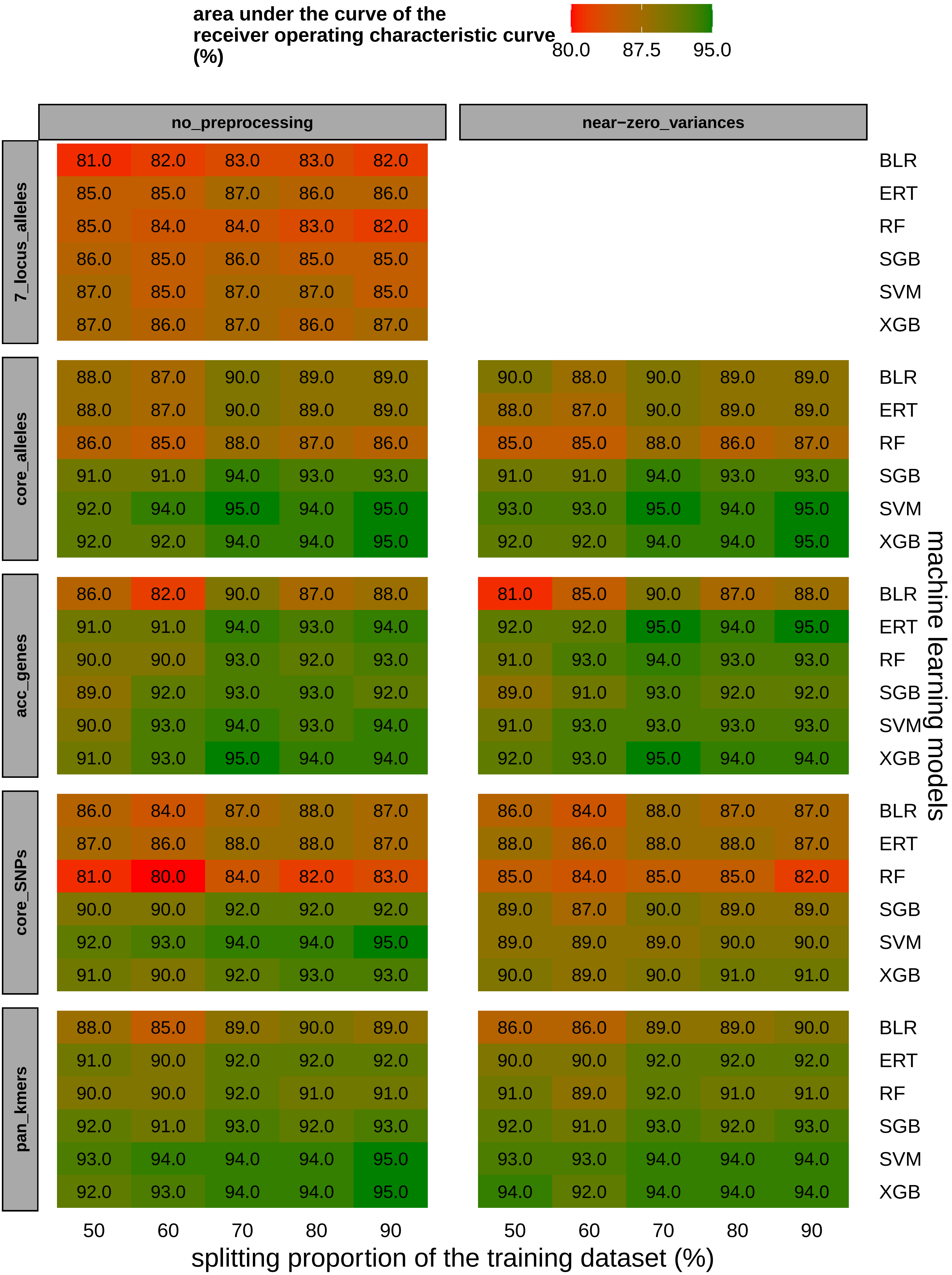
area under the curve of the receiver operating characteristic curve (%)
80.0
87.5
95.0
no_preprocessing
near−zero_variances
7_locus_alleles
BLR 81.0 82.0 83.0 83.0 82.0
ERT 85.0 85.0 87.0 86.0 86.0
RF 85.0 84.0 84.0 83.0 82.0
SGB 86.0 85.0 86.0 85.0 85.0
SVM 87.0 85.0 87.0 87.0 85.0
XGB 87.0 86.0 87.0 86.0 87.0
core_alleles
BLR 88.0 87.0 90.0 89.0 89.0 | 90.0 88.0 90.0 89.0 89.0
ERT 88.0 87.0 90.0 89.0 89.0 | 88.0 87.0 90.0 89.0 89.0
RF 86.0 85.0 88.0 87.0 86.0 | 85.0 85.0 88.0 86.0 87.0
SGB 91.0 91.0 94.0 93.0 93.0 | 91.0 91.0 94.0 93.0 93.0
SVM 92.0 94.0 95.0 94.0 95.0 | 93.0 93.0 95.0 94.0 95.0
XGB 92.0 92.0 94.0 94.0 95.0 | 92.0 92.0 94.0 94.0 95.0
acc_genes
BLR 86.0 82.0 90.0 87.0 88.0 | 81.0 85.0 90.0 87.0 88.0
ERT 91.0 91.0 94.0 93.0 94.0 | 92.0 92.0 95.0 94.0 95.0
RF 90.0 90.0 93.0 92.0 93.0 | 91.0 93.0 94.0 93.0 93.0
SGB 89.0 92.0 93.0 93.0 92.0 | 89.0 91.0 93.0 92.0 92.0
SVM 90.0 93.0 94.0 93.0 94.0 | 91.0 93.0 93.0 93.0 93.0
XGB 91.0 93.0 95.0 94.0 94.0 | 92.0 93.0 95.0 94.0 94.0
core_SNPs
BLR 86.0 84.0 87.0 88.0 87.0 | 86.0 84.0 88.0 87.0 87.0
ERT 87.0 86.0 88.0 88.0 87.0 | 88.0 86.0 88.0 88.0 87.0
RF 81.0 80.0 84.0 82.0 83.0 | 85.0 84.0 85.0 85.0 82.0
SGB 90.0 90.0 92.0 92.0 92.0 | 89.0 87.0 90.0 89.0 89.0
SVM 92.0 93.0 94.0 94.0 95.0 | 89.0 89.0 89.0 90.0 90.0
XGB 91.0 90.0 92.0 93.0 93.0 | 90.0 89.0 90.0 91.0 91.0
pan_kmers
BLR 88.0 85.0 89.0 90.0 89.0 | 86.0 86.0 89.0 89.0 90.0
ERT 91.0 90.0 92.0 92.0 92.0 | 90.0 90.0 92.0 92.0 92.0
RF 90.0 90.0 92.0 91.0 91.0 | 91.0 89.0 92.0 91.0 91.0
SGB 92.0 91.0 93.0 92.0 93.0 | 92.0 91.0 93.0 92.0 93.0
SVM 93.0 94.0 94.0 94.0 95.0 | 93.0 93.0 94.0 94.0 94.0
XGB 92.0 93.0 94.0 94.0 95.0 | 94.0 92.0 94.0 94.0 94.0
50 60 70 80 90 | 50 60 70 80 90
splitting proportion of the training dataset (%)
machine learning models

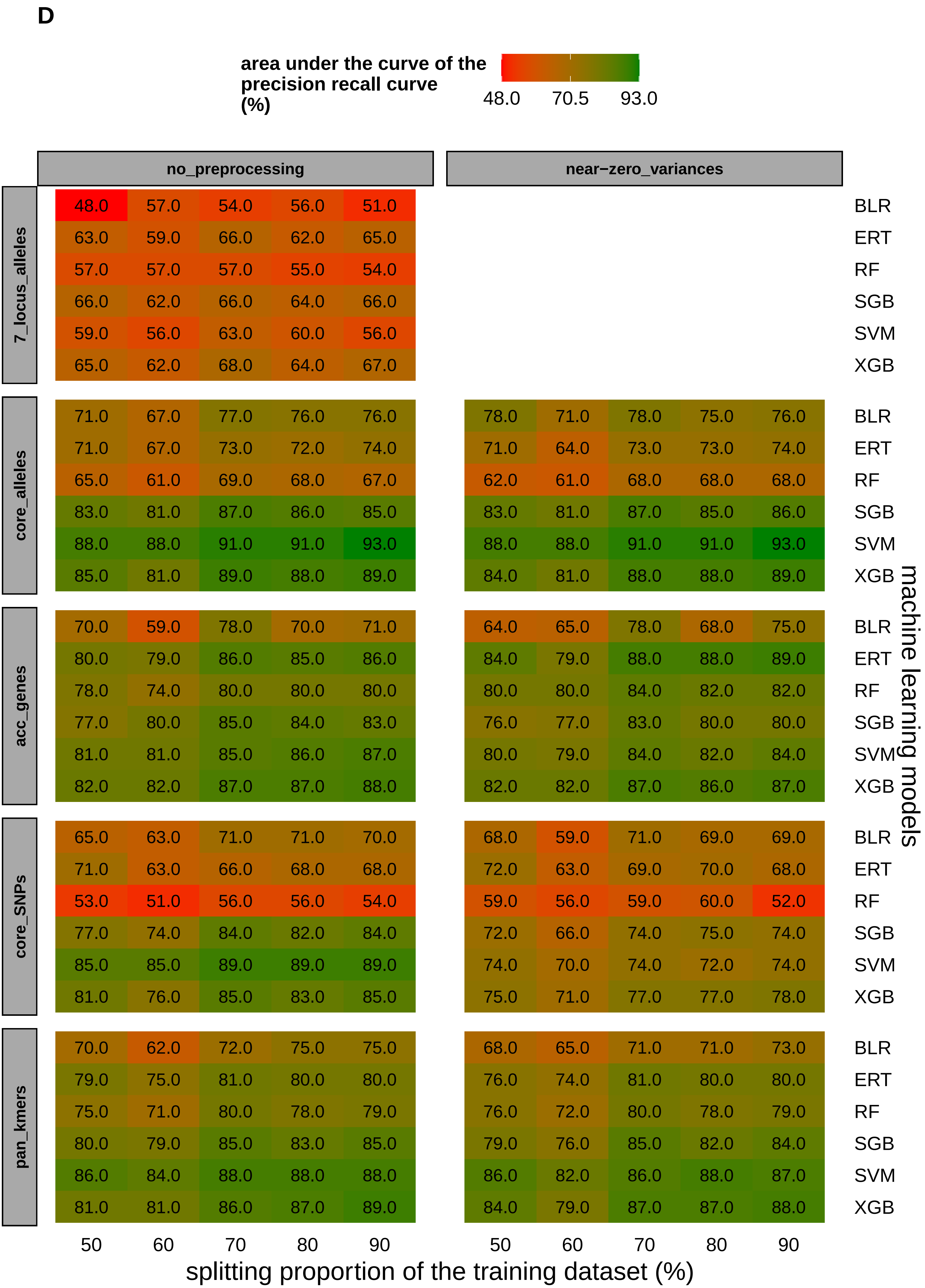
D
area under the curve of the precision recall curve (%)
48.0
70.5
93.0
no_preprocessing
near−zero_variances
7_locus_alleles
BLR 48.0 57.0 54.0 56.0 51.0
ERT 63.0 59.0 66.0 62.0 65.0
RF 57.0 57.0 57.0 55.0 54.0
SGB 66.0 62.0 66.0 64.0 66.0
SVM 59.0 56.0 63.0 60.0 56.0
XGB 65.0 62.0 68.0 64.0 67.0
core_alleles
BLR 71.0 67.0 77.0 76.0 76.0 | 78.0 71.0 78.0 75.0 76.0
ERT 71.0 67.0 73.0 72.0 74.0 | 71.0 64.0 73.0 73.0 74.0
RF 65.0 61.0 69.0 68.0 67.0 | 62.0 61.0 68.0 68.0 68.0
SGB 83.0 81.0 87.0 86.0 85.0 | 83.0 81.0 87.0 85.0 86.0
SVM 88.0 88.0 91.0 91.0 93.0 | 88.0 88.0 91.0 91.0 93.0
XGB 85.0 81.0 89.0 88.0 89.0 | 84.0 81.0 88.0 88.0 89.0
acc_genes
BLR 70.0 59.0 78.0 70.0 71.0 | 64.0 65.0 78.0 68.0 75.0
ERT 80.0 79.0 86.0 85.0 86.0 | 84.0 79.0 88.0 88.0 89.0
RF 78.0 74.0 80.0 80.0 80.0 | 80.0 80.0 84.0 82.0 82.0
SGB 77.0 80.0 85.0 84.0 83.0 | 76.0 77.0 83.0 80.0 80.0
SVM 81.0 81.0 85.0 86.0 87.0 | 80.0 79.0 84.0 82.0 84.0
XGB 82.0 82.0 87.0 87.0 88.0 | 82.0 82.0 87.0 86.0 87.0
core_SNPs
BLR 65.0 63.0 71.0 71.0 70.0 | 68.0 59.0 71.0 69.0 69.0
ERT 71.0 63.0 66.0 68.0 68.0 | 72.0 63.0 69.0 70.0 68.0
RF 53.0 51.0 56.0 56.0 54.0 | 59.0 56.0 59.0 60.0 52.0
SGB 77.0 74.0 84.0 82.0 84.0 | 72.0 66.0 74.0 75.0 74.0
SVM 85.0 85.0 89.0 89.0 89.0 | 74.0 70.0 74.0 72.0 74.0
XGB 81.0 76.0 85.0 83.0 85.0 | 75.0 71.0 77.0 77.0 78.0
pan_kmers
BLR 70.0 62.0 72.0 75.0 75.0 | 68.0 65.0 71.0 71.0 73.0
ERT 79.0 75.0 81.0 80.0 80.0 | 76.0 74.0 81.0 80.0 80.0
RF 75.0 71.0 80.0 78.0 79.0 | 76.0 72.0 80.0 78.0 79.0
SGB 80.0 79.0 85.0 83.0 85.0 | 79.0 76.0 85.0 82.0 84.0
SVM 86.0 84.0 88.0 88.0 88.0 | 86.0 82.0 86.0 88.0 87.0
XGB 81.0 81.0 86.0 87.0 89.0 | 84.0 79.0 87.0 87.0 88.0
50 60 70 80 90 | 50 60 70 80 90
splitting proportion of the training dataset (%)
machine learning models

E

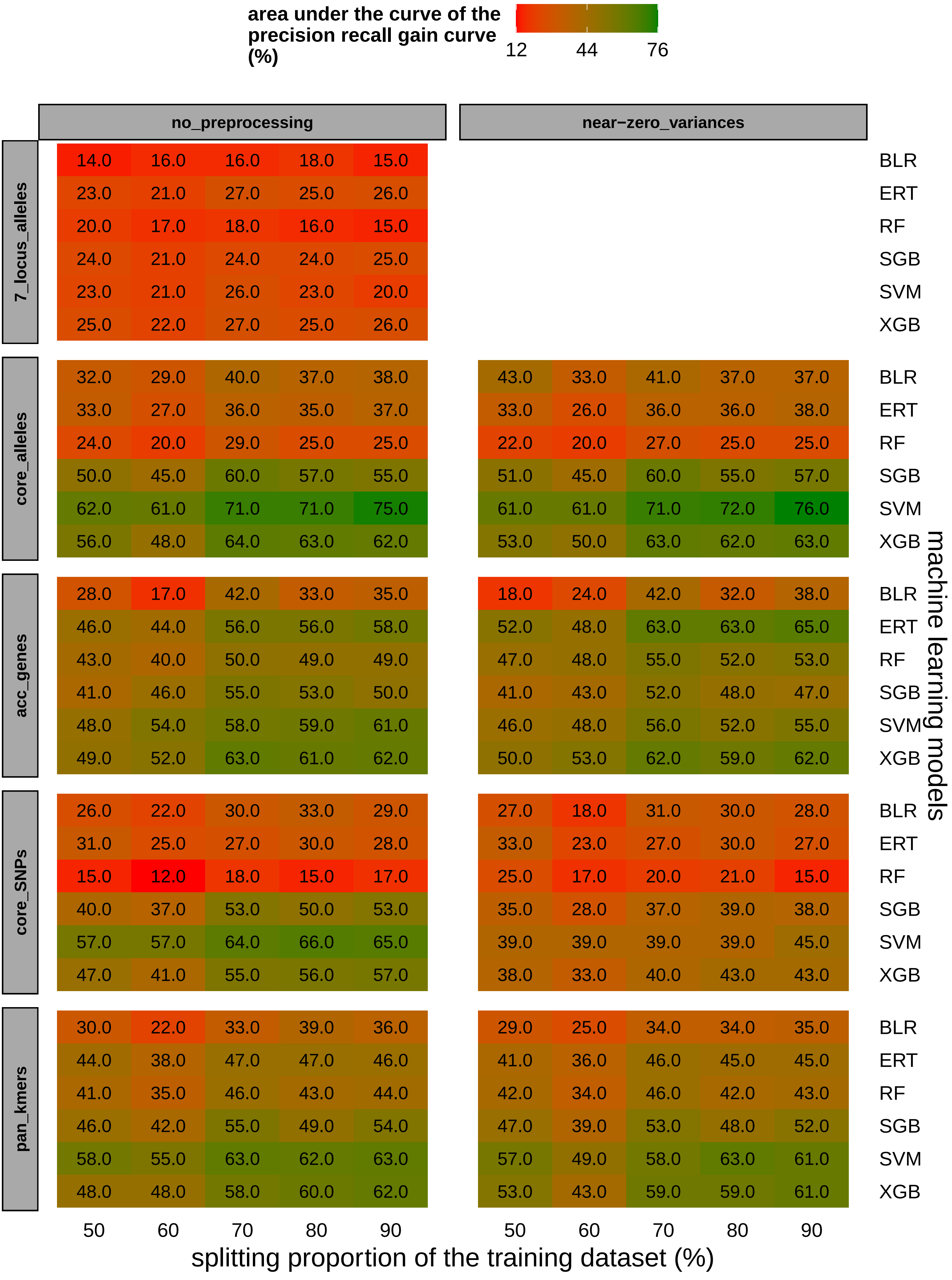

area under the curve of the precision recall gain curve (%)
12
44
76
no_preprocessing
near−zero_variances
7_locus_alleles
14.0 16.0 16.0 18.0 15.0 BLR
23.0 21.0 27.0 25.0 26.0 ERT
20.0 17.0 18.0 16.0 15.0 RF
24.0 21.0 24.0 24.0 25.0 SGB
23.0 21.0 26.0 23.0 20.0 SVM
25.0 22.0 27.0 25.0 26.0 XGB
core_alleles
32.0 29.0 40.0 37.0 38.0 43.0 33.0 41.0 37.0 37.0 BLR
33.0 27.0 36.0 35.0 37.0 33.0 26.0 36.0 36.0 38.0 ERT
24.0 20.0 29.0 25.0 25.0 22.0 20.0 27.0 25.0 25.0 RF
50.0 45.0 60.0 57.0 55.0 51.0 45.0 60.0 55.0 57.0 SGB
62.0 61.0 71.0 71.0 75.0 61.0 61.0 71.0 72.0 76.0 SVM
56.0 48.0 64.0 63.0 62.0 53.0 50.0 63.0 62.0 63.0 XGB
acc_genes
28.0 17.0 42.0 33.0 35.0 18.0 24.0 42.0 32.0 38.0 BLR
46.0 44.0 56.0 56.0 58.0 52.0 48.0 63.0 63.0 65.0 ERT
43.0 40.0 50.0 49.0 49.0 47.0 48.0 55.0 52.0 53.0 RF
41.0 46.0 55.0 53.0 50.0 41.0 43.0 52.0 48.0 47.0 SGB
48.0 54.0 58.0 59.0 61.0 46.0 48.0 56.0 52.0 55.0 SVM
49.0 52.0 63.0 61.0 62.0 50.0 53.0 62.0 59.0 62.0 XGB
core_SNPs
26.0 22.0 30.0 33.0 29.0 27.0 18.0 31.0 30.0 28.0 BLR
31.0 25.0 27.0 30.0 28.0 33.0 23.0 27.0 30.0 27.0 ERT
15.0 12.0 18.0 15.0 17.0 25.0 17.0 20.0 21.0 15.0 RF
40.0 37.0 53.0 50.0 53.0 35.0 28.0 37.0 39.0 38.0 SGB
57.0 57.0 64.0 66.0 65.0 39.0 39.0 39.0 39.0 45.0 SVM
47.0 41.0 55.0 56.0 57.0 38.0 33.0 40.0 43.0 43.0 XGB
pan_kmers
30.0 22.0 33.0 39.0 36.0 29.0 25.0 34.0 34.0 35.0 BLR
44.0 38.0 47.0 47.0 46.0 41.0 36.0 46.0 45.0 45.0 ERT
41.0 35.0 46.0 43.0 44.0 42.0 34.0 46.0 42.0 43.0 RF
46.0 42.0 55.0 49.0 54.0 47.0 39.0 53.0 48.0 52.0 SGB
58.0 55.0 63.0 62.0 63.0 57.0 49.0 58.0 63.0 61.0 SVM
48.0 48.0 58.0 60.0 62.0 53.0 43.0 59.0 59.0 61.0 XGB
50 60 70 80 90 50 60 70 80 90
splitting proportion of the training dataset (%)
machine learning models

F

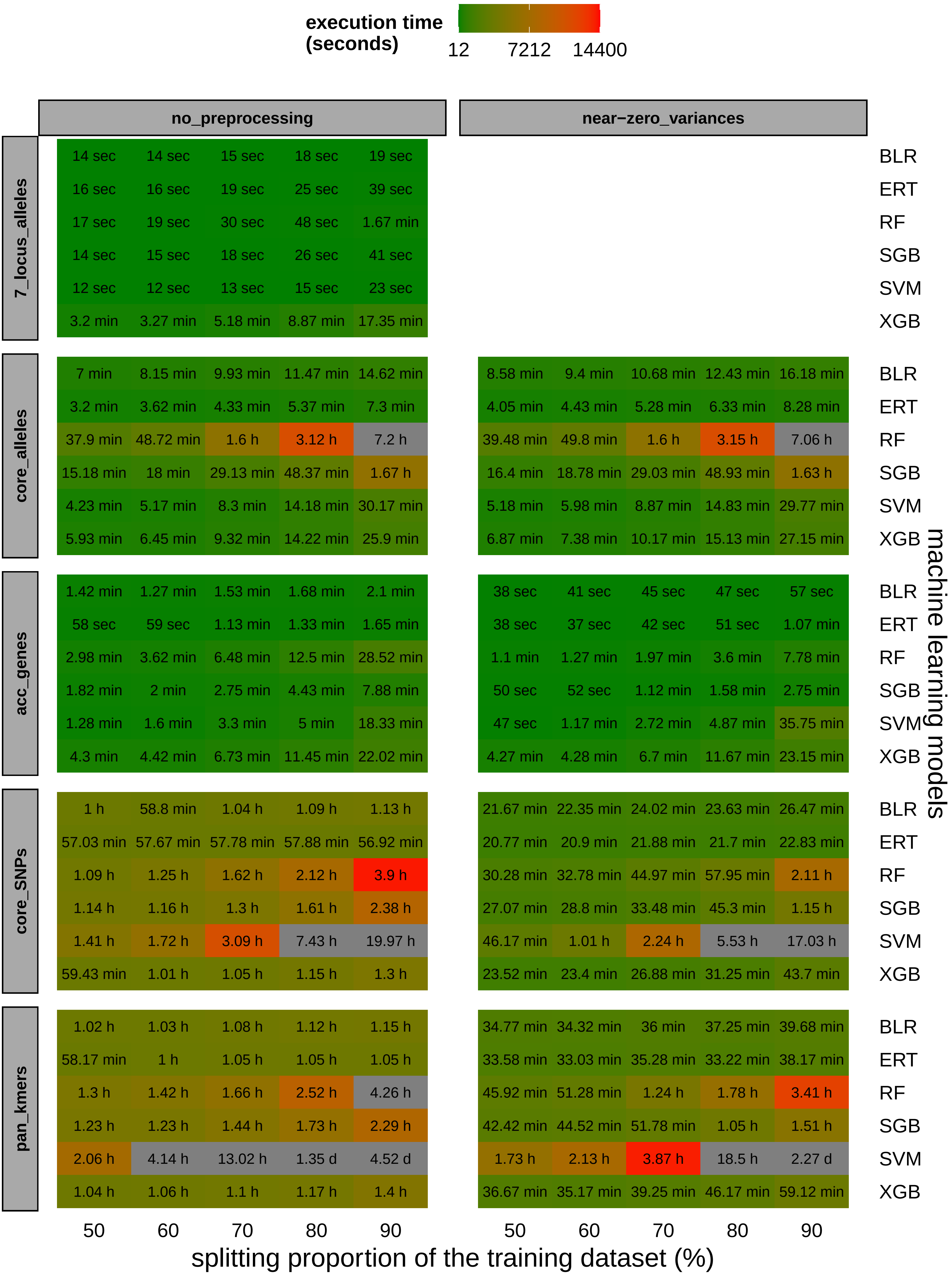

execution time
(seconds)
12
7212
14400
no_preprocessing
near−zero_variances
7_locus_alleles
14 sec 14 sec 15 sec 18 sec 19 sec
16 sec 16 sec 19 sec 25 sec 39 sec
17 sec 19 sec 30 sec 48 sec 1.67 min
14 sec 15 sec 18 sec 26 sec 41 sec
12 sec 12 sec 13 sec 15 sec 23 sec
3.2 min 3.27 min 5.18 min 8.87 min 17.35 min
core_alleles
7 min 8.15 min 9.93 min 11.47 min 14.62 min
3.2 min 3.62 min 4.33 min 5.37 min 7.3 min
37.9 min 48.72 min 1.6 h 3.12 h 7.2 h
15.18 min 18 min 29.13 min 48.37 min 1.67 h
4.23 min 5.17 min 8.3 min 14.18 min 30.17 min
5.93 min 6.45 min 9.32 min 14.22 min 25.9 min
8.58 min 9.4 min 10.68 min 12.43 min 16.18 min
4.05 min 4.43 min 5.28 min 6.33 min 8.28 min
39.48 min 49.8 min 1.6 h 3.15 h 7.06 h
16.4 min 18.78 min 29.03 min 48.93 min 1.63 h
5.18 min 5.98 min 8.87 min 14.83 min 29.77 min
6.87 min 7.38 min 10.17 min 15.13 min 27.15 min
acc_genes
1.42 min 1.27 min 1.53 min 1.68 min 2.1 min
58 sec 59 sec 1.13 min 1.33 min 1.65 min
2.98 min 3.62 min 6.48 min 12.5 min 28.52 min
1.82 min 2 min 2.75 min 4.43 min 7.88 min
1.28 min 1.6 min 3.3 min 5 min 18.33 min
4.3 min 4.42 min 6.73 min 11.45 min 22.02 min
38 sec 41 sec 45 sec 47 sec 57 sec
38 sec 37 sec 42 sec 51 sec 1.07 min
1.1 min 1.27 min 1.97 min 3.6 min 7.78 min
50 sec 52 sec 1.12 min 1.58 min 2.75 min
47 sec 1.17 min 2.72 min 4.87 min 35.75 min
4.27 min 4.28 min 6.7 min 11.67 min 23.15 min
core_SNPs
1 h 58.8 min 1.04 h 1.09 h 1.13 h
57.03 min 57.67 min 57.78 min 57.88 min 56.92 min
1.09 h 1.25 h 1.62 h 2.12 h 3.9 h
1.14 h 1.16 h 1.3 h 1.61 h 2.38 h
1.41 h 1.72 h 3.09 h 7.43 h 19.97 h
59.43 min 1.01 h 1.05 h 1.15 h 1.3 h
21.67 min 22.35 min 24.02 min 23.63 min 26.47 min
20.77 min 20.9 min 21.88 min 21.7 min 22.83 min
30.28 min 32.78 min 44.97 min 57.95 min 2.11 h
27.07 min 28.8 min 33.48 min 45.3 min 1.15 h
46.17 min 1.01 h 2.24 h 5.53 h 17.03 h
23.52 min 23.4 min 26.88 min 31.25 min 43.7 min
pan_kmers
1.02 h 1.03 h 1.08 h 1.12 h 1.15 h
58.17 min 1 h 1.05 h 1.05 h 1.05 h
1.3 h 1.42 h 1.66 h 2.52 h 4.26 h
1.23 h 1.23 h 1.44 h 1.73 h 2.29 h
2.06 h 4.14 h 13.02 h 1.35 d 4.52 d
1.04 h 1.06 h 1.1 h 1.17 h 1.4 h
34.77 min 34.32 min 36 min 37.25 min 39.68 min
33.58 min 33.03 min 35.28 min 33.22 min 38.17 min
45.92 min 51.28 min 1.24 h 1.78 h 3.41 h
42.42 min 44.52 min 51.78 min 1.05 h 1.51 h
1.73 h 2.13 h 3.87 h 18.5 h 2.27 d
36.67 min 35.17 min 39.25 min 46.17 min 59.12 min
BLR
ERT
RF
SGB
SVM
XGB
machine learning models
50 60 70 80 90
50 60 70 80 90
splitting proportion of the training dataset (%)
